# Inanspruchnahme psychiatrisch-psychotherapeutischer Versorgung in Deutschland während des ersten Jahres der COVID-19-Pandemie – systematischer Review und Metaanalyse

**DOI:** 10.1007/s00115-025-01812-y

**Published:** 2025-03-07

**Authors:** Antranik Erdekian, Miriam Glock, Sophia Huetter, Mike Rueb, Dirk Riedinger, Jutta Stoffers-Winterling, Saskia Lindner, Fabian Baum, Lars P. Hölzel, Oliver Tüscher, Klaus Lieb, Kristina Adorjan, Hauke Felix Wiegand

**Affiliations:** 1 Universitätsklinik und Poliklinik für Psychiatrie, Psychotherapie und Psychosomatik, Universitätsmedizin Halle, Julius-Kühn-Straße 7, 06112 Halle (Saale), Deutschland; 2 Klinik für Psychiatrie und Psychotherapie, Universitätsmedizin der Johannes Guttenberg Universität Mainz, Mainz, Deutschland; 3 Klinik für Psychiatrie und Psychotherapie, LMU Klinikum, Ludwig Maximilians Universität München, München, Deutschland; 4 Leibniz-Institut für Resilienzforschung, Mainz, Deutschland; 5 Zentrum für Evidenzbasierte Gesundheitsversorgung, Universitätsklinikum und Medizinische Fakultät Carl Gustav Carus an der Technischen Universität Dresden, Dresden, Deutschland; 6 Oberberg Parkklinik Wiesbaden Schlangenbad, Schlangenbad, Deutschland; 7 Klinik für Psychiatrie und Psychotherapie, Universität Bern, Bern, Schweiz

**Keywords:** Psychiatrische Notfallversorgung, Stationäre Versorgung, Ambulante Versorgung, Psychotrope Medikamente, Pandemiejahr 2020, Psychiatric emergency care, Inpatient treatment, Outpatient treatment, Psychotropic drugs, Pandemic year 2020

## Abstract

**Hintergrund:**

In der COVID-19(„coronavirus disease 2019“)-Pandemie zeigten Studien Hinweise auf Veränderungen der Inanspruchnahme der stationären und ambulanten psychiatrisch-psychotherapeutischen Versorgung sowie der psychiatrischen Notfallversorgung. Beobachtungsebene und Repräsentativität dieser Studien waren jedoch heterogen.

**Ziele der Arbeit:**

Veränderungen der Inanspruchnahme psychiatrisch-psychotherapeutischer Versorgung im ersten Jahr der COVID-19-Pandemie wurden durch systematische Literatursuche, Bewertung der Qualitäts- und Beobachtungsebene sowie Metaanalyse der Effekte eingeordnet.

**Material und Methoden:**

Systematische Suche in PubMed, PsycInfo und Embase bis Juni 2023 sowie Nachsuche in PubMed bis einschließlich Oktober 2024. Daten wurden den Zeiträumen 1. Lockdownphase, Zwischenlockdownphase, 2. Lockdownphase, ganzes Pandemiejahr 2020 zugeordnet.

**Ergebnisse:**

Insgesamt konnten 17 Studien eingeschlossen werden. Es zeigten sich für die Anzahl stationärer Aufnahmen Reduktionen für die 1. Lockdownphase von RR 0,74; 95 %-KI [0,70; 0,79]; I^2^ 95,5 %; t^2^ 0,0053 und für die 2. Lockdownphase von RR 0,78; 95 %-KI [0,75; 0,81]; I^2^ 97,1 %; t^2^ 0,0058. Für psychiatrische Notfallversorgung wurden nur Studien niedriger Beobachtungsebene gefunden und für ambulante Inanspruchnahme nur zwei Studien mit unterschiedlichen Indikatoren. Bezüglich der Verordnung psychotroper Medikamente zeigten sich keine eindeutigen Veränderungen.

**Diskussion:**

Im ersten Jahr der COVID-19-Pandemie war die Inanspruchnahme psychiatrisch-psychotherapeutischer Versorgung insbesondere für den stationären Sektor reduziert. Die Auswirkungen dieser signifikanten Einschränkungen sind unklar. Wir schlagen daher eine Versorgungs-Surveillance vor, die solche Veränderungen und mögliche Folgen zeitnah erfassen könnte.

**Zusatzmaterial online:**

Die Online-Version dieses Beitrags (10.1007/s00115-025-01812-y) enthält Tabellen und weiteres Zusatzmaterial.

## Abstract (en)

**Background:**

During the coronavirus disease 2019 (COVID-19) pandemic studies showed indications of changes in the utilization of inpatient, outpatient and emergency mental healthcare services; however, the level of observation and representativeness of these studies were heterogeneous.

**Objectives:**

Changes in the utilization of psychiatric and psychotherapeutic care in the first year of the COVID-19 pandemic were classified through a systematic literature search, evaluation of the quality and observational levels as well as a meta-analysis of the effects.

**Material and methods:**

A systematic search was carried out in PubMed, PsycInfo and Embase until June 2023 and a follow-up search in PubMed up to and including October 2024. Data were assigned to the periods first lockdown phase, between lockdown phases, second lockdown phase and entire pandemic year 2020.

**Results:**

A total of 17 studies were included. There were reductions in the number of inpatient admissions for the first lockdown phase of relative risk, RR 0.74, 95% confidence interval CI 0.70–0.79, I^2^ 95.5%, t^2^ 0.0053 and for the second lockdown phase of RR 0.78, 95% CI 0.75–0.81, I^2^ 97.1%, t^2^ 0.0058. Only studies with a low observational level were found for psychiatric emergency care and only two studies with different indicators for outpatient utilization. There were no clear changes in the prescription of psychotropic drugs.

**Conclusion:**

In the first year of the COVID-19 pandemic utilization of mental healthcare services was reduced, especially for the inpatient sector. The effects of these significant restrictions are unclear. We therefore propose a mental healthcare surveillance that could promptly record such changes and possible consequences.

## Hintergrund

Die COVID-19(„coronavirus disease 2019“)-Pandemie stellte eine große Herausforderung für die psychiatrisch-psychotherapeutische Versorgung dar. Das Angebot von Behandlungskapazitäten war aus mehreren Gründen einem Veränderungsdruck ausgesetzt: Infektionsschutzmaßnahmen mussten in Settings umgesetzt werden, die oft eher auf Kontakte und Gemeinschaft ausgelegt waren. Kapazitäten für Menschen mit schweren Antriebsstörungen, Desorganisationssyndromen oder akuter Suizidalität aufgrund psychischer Erkrankungen und komorbider SARS-CoV-2(„severe acute respiratory syndrome coronavirus type 2“)-Infektion mussten vorgehalten werden, da diese Populationen nicht auf regulären Infektionsstationen versorgt werden konnten. Zudem wurden seitens der Gesundheitspolitik zeitweise die Anreize des Vergütungssystems so verändert, dass die Nichtbelegung stationärer Kapazitäten zu bevorzugen war (sog. „Freihalteprämien“). Zugleich waren im ambulanten System zeitweise telemedizinische Leistungen einfacher umsetzbar. Auch die Nachfrage unterlag potenziell Veränderungen: Menschen fürchteten Infektionen in Einrichtungen des Gesundheitssystems und vermieden möglicherweise ambulante Termine und Krankenhausaufenthalte [24]. Es trat nicht die befürchtete Welle an psychischen Erkrankungen ein [21] und ein Großteil der Bevölkerung erwies sich als resilient, wobei es auch Hinweise auf eine Zunahme der psychischen Belastung im Laufe der Pandemie gab [20]. Und gerade für Menschen mit Einschränkungen durch psychische Erkrankungen waren viele komplementäre Angebote wie Tagesstätten und Werkstätten zeitweise nicht verfügbar [10].

Frühe Umfragestudien wiesen auf signifikante Einschränkungen der Inanspruchnahme im stationären und teilstationären [2, 24] sowie im ambulanten psychiatrisch-psychotherapeutischen [10] Versorgungssystem hin. Auch zeigten Berichte zur Versorgung psychiatrischer Notfälle in einzelnen Notaufnahmen Veränderungen an [18]. Zudem gab es Hinweise, dass Populationen mit bestimmten Diagnosegruppen ein unterschiedliches Ausmaß von Reduktionen der Inanspruchnahme aufwiesen [10, 24]. Viele repräsentative Studien in Krankenkassenroutinedaten wurden aufgrund der Zugangshürden und Zeitlatenz der Datenbereitstellung aber erst verzögert veröffentlicht, sodass auch eine reliable Beurteilung der Veränderungen im ersten Jahr der COVID-19-Pandemie erst jetzt erfolgen kann. Ein systematischer Überblick zum Ausmaß der Einschränkungen und zu betroffenen Bereichen und Populationen ist jedoch von großer Relevanz, um aus der Krisenlage der Pandemie Lehren ziehen zu können und im Sinne der „crisis preparedness“ auf zukünftige Krisen vorbereitet zu sein.

Ziel dieser Studie waren daher ein systematischer Review und – sofern möglich – eine Metaanalyse zu der Frage, wie sich die Inanspruchnahme psychiatrisch-psychotherapeutischer Versorgung im ersten Jahr der COVID-19-Pandemie und insbesondere während der beiden Lockdownphasen in den Bereichen stationärer Versorgung, psychiatrischer Notfallversorgung, ambulanter Versorgung und Verordnung ambulanter psychotroper Medikation in Deutschland im Vergleich zu Zeitintervallen vor der Pandemie verändert hat.

## Material und Methoden

### Einschluss- und Ausschlusskriterien

Es wurden nur Studien eingeschlossen, die die Auswirkungen der COVID-19-Pandemie bzw. der damit verbundenen Schutzmaßnahmen (*Exposure) *auf die Inanspruchnahme psychiatrischer, psychosomatischer oder psychotherapeutischer Versorgungsleistungen des Deutschen Gesundheitssystems (*Outcome*) untersuchten. Bezüglich der *Population *wurden nur Erwachsene ab 18 Jahren mit einer diagnostizierten psychischen Erkrankung nach ICD-10 (International Statistical Classification of Diseases and Related Health Problems 10) Kapitel F eingeschlossen. Es wurden nur Routinedatenstudien, retro- und prospektive Beobachtungsstudien (z. B. Kohortenstudien, Fall-Kontroll-Studien), Querschnittsstudien und quantitative Umfragestudien sowie Studien mit einer vergleichenden oder längsschnittlichen Analyse einbezogen (*Studiendesign*). Als *Outcome* mussten die Studien allgemein die Inanspruchnahme stationärer und/oder ambulanter psychiatrischer, psychosomatischer und psychotherapeutischer Leistungen, Veränderungen der Inanspruchnahme innerhalb bestimmter Settings, Veränderungen der Inanspruchnahme innerhalb spezifischer Diagnosegruppen sowie Veränderungen des Einsatzes psychotroper Medikamente untersuchen.

Studien wurden ausgeschlossen, wenn sie nicht auf das deutsche Gesundheitssystem bezogen waren, zu mehr als 15 % des Gesamtsamples und nicht abgrenzbar andere Altersgruppen als Erwachsene berücksichtigten, ein interventionelles Studiendesign, nur geschätzte Zahlen (z. B. in Surveys) oder Fallberichte beinhalteten oder sich ausschließlich auf nichtpsychiatrische Gesundheitsleistungen fokussierten.

Es wurden potenziell nur Studien in deutscher, englischer oder französischer Sprache ausgewählt.

### Suchstrategie und Screening

**Abb. 1:**
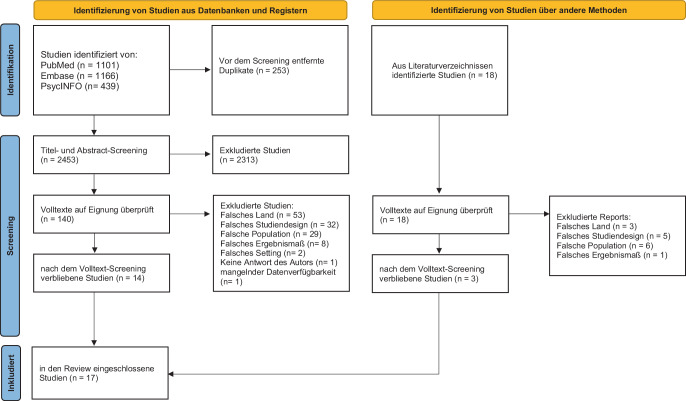
PRISMA-Flussdiagramm: Suche, Screening und Einschluss der Studien

Das Studienprotokoll wurde bei Prospero registriert (CRD42023441542; [6]). Die Suchstrategie kombinierte durch die logische Verknüpfung mit „OR“ Themenblöcke zu COVID-19 (z. B. „coronavirus“ oder „sars cov 2“), psychischen Erkrankungen (z. B. „mental illness“ oder „mental health“) sowie der Inanspruchnahme von Gesundheitsleistungen (z. B. „admissions“ oder „inpatient“; Details online in Supplement A). Die initiale Suche in PubMed, PsycInfo und Embase fand am 13/08/2023 statt. Hierbei wurden Studien identifiziert, die zwischen 12/2020 und 08/2023 publiziert wurden. Im Oktober 2024 wurde in PubMed eine Aktualisierungssuche durchgeführt, bei der noch zwischen 09/2023 und 09/2024 veröffentlichte Studien identifiziert wurden. Zudem wurden einerseits die Referenzlisten bei der Suche gefundener Reviews manuell auf weitere, bisher nicht identifizierte Studien durchsucht und andererseits uns bekannte Berichte von Körperschaften des Gesundheitssystems (z. B. Berichte der Kassenärztliche Vereinigung, Krankenhausreport [4, 17]) nach relevanten Informationen durchsucht und, wenn geeignet, in die Analyse einbezogen (Abb. 1).

Die identifizierten Referenzen wurden zunächst in EndNote (®Clarivate, Phildalephia, Pennsylvania, Vereinigte Staaten, and London, Vereinigtes Königreich) importiert und dort auf Duplikate geprüft. Anschließend wurden mithilfe von Covidence (®Veritas Health Innovation, Clarivate) Titel und Abstracts der gefundenen Studien jeweils durch zwei unabhängige Reviewer (A. E., M. G., M. R., D. R. und S. H.) gescreent. Die hier selektierten Studien wurden dann in der Volltextprüfung wiederum von zwei unabhängigen Reviewern (A. E., M. G.) anhand der Ein- und Ausschlusskriterien bewertet und die Daten extrahiert. Bei Unstimmigkeiten wurde ein Konsens durch Diskussion oder die Hinzuziehung eines dritten Reviewers (H. F. W.) erreicht.

### Datenextraktion

Aus den eingeschlossenen Studien wurden folgende Daten erfasst:

- Studiendesign,
- Studienzeitraum,
- Studiensetting (z. B. ambulant, stationär, Notaufnahme),
- Populationsmerkmale (Alter, Geschlecht),
- Stichprobengröße (Anzahl der Patienten) sowie
- die Outcomes.

Die Outcomes umfassten psychiatrische Diagnosen, die Anzahl der Aufnahmen, Verweildauern, Kontakte, ambulante Medikation nach „defined daily doses“ (DDD; [26]) im zeitlichen Verlauf und ggf. aufgeschlüsselt nach ICD-10-Diagnosegruppen.

### Studienzeiträume

Die in den Studien berichteten Zeiträume wurden folgendermaßen kategorisiert:

- 1. Lockdown (März bis Mai 2020),
- 2. Lockdown (Dezember 2020 bis Februar 2021),
- die Zwischenphase (Juni bis November 2020) und
- das gesamte Jahr 2020.

### Beobachtungsebene der Studien

Die Studien wurden zusätzlich anhand der Beobachtungsebene in drei Kategorien eingeteilt, um die quantitativen Veränderungen der Inanspruchnahme während der COVID-19-Pandemie verlässlicher abschätzen zu können:

- Kategorie A umfasste Studien mit versorgungsnahen Daten der Krankenkassen aus mindestens der Hälfte der Bundesländer.
- Kategorie B umfasste Studien, die Daten von mehreren Gesundheitseinrichtungen zusammenfassten.
- Kategorie C umfasste Studien aus nur einer Gesundheitseinrichtung.

### Qualitätsbewertung

Die Qualität der eingeschlossenen Studien wurde anhand einer modifizierten Version der Newcastle-Ottawa-Skala (NOS) für Kohortenstudien bewertet [23]. Diese modifizierte Skala bewertet die Studien anhand der drei Hauptkategorien Selektion der Stichproben, Vergleichbarkeit und Endpunkterfassung. Ein Maximum von 7 Sternen kann vergeben werden. Die verwendete Skala und die Ergebnisse sind online im Supplement B hinterlegt.

### Datenanalyse

Die Metaanalyse wurde mit R Studio (Version 2024.04.2 + 764) mit einem Random-Effects-Modell durchgeführt (meta und metafor Packages), da die Einzelstudien unterschiedliche, aber miteinander verbundene Ereigniseffekte (COVID-19-Pandemie) aufwiesen, und um die Heterogenität der Studien adäquat zu berücksichtigen [13]. Als Maße der Heterogenität wurde die I^2^- und τ^2^-Statistik verwendet. Die Heterogenität wurde anhand der I^2^-Statistik aus Cochran’s Q und τ^2^ bewertet, berechnet mit der Restricted-Maximum-Likelihood(REML)-Methode.

## Ergebnisse

Es wurden initial 2706 Studien in den drei Datenbanken gefunden, wovon nach Entfernung von 253 Duplikaturen 2453 Studien blieben. Nach Titel- und Abstracts-Screening verblieben 140 Studien. 18 weitere Studien wurden durch Suche in den Literaturverzeichnissen identifiziert. Nach dem Volltextscreening blieben 18 Studien. Eine geeignete Studie [5] musste wegen Nichtverfügbarkeit der Daten ausgeschlossen werden (Abb. 1).

Insgesamt wurden 17 Studien in die Metaanalyse eingeschlossen (Tab. e1 online): 7 Studien untersuchten den stationären Sektor, 7 Studien befassten sich mit der Versorgung in der Notaufnahme, 2 Studien untersuchten die ambulante Versorgung und 4 Studien beschäftigten sich mit ambulanten Verordnungen psychotroper Medikation. Anhand der I^2^- und τ^2^-Statistik ließ sich über alle Auswertungen eine große Heterogenität feststellen. Analysen für Diagnosesubgruppen waren nur für den stationären Sektor möglich, da nur hier in Anzahl, Qualität und Repräsentativität ausreichend Studien vorlagen.

### Stationärer Sektor

**Abb. 2:**
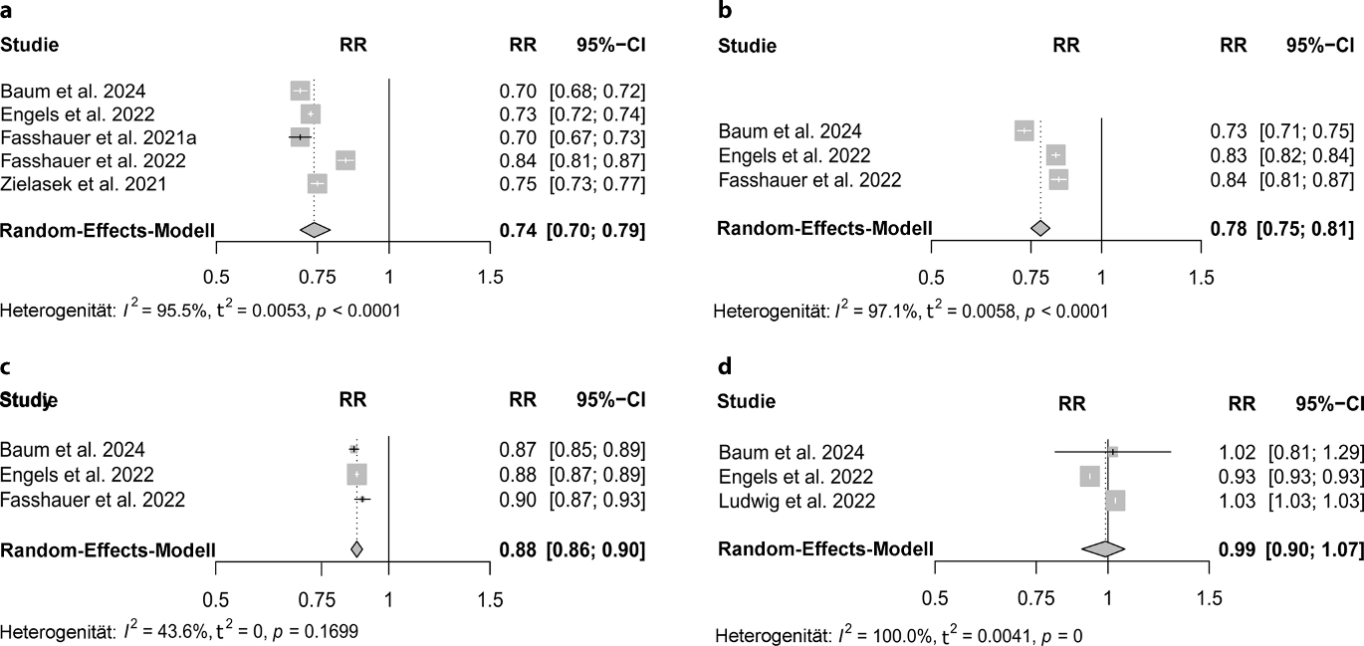
Stationäre Aufnahmen – alle Diagnosen. **a** 1. Lockdownphase, **b** 2. Lockdownphase,** c** Zwischenlockdownphase,** d** ambulante Medikation („defined daily doses“, DDD) das ganze Jahr 2020. *RR* Relatives Risiko, *95%-CI* 95 %-Konfidenzintervall

Insgesamt untersuchten 7 A- und B‑Studien mit hoher Qualität (NOS 6/7 Sterne) die Veränderung der Anzahl stationärer Aufnahmen während der COVID-19-Pandemie im Vergleich zum Vorjahr. Es zeigt sich eine signifikante Verringerung der stationären Inanspruchnahme (Tab. e2 online) für die 1. Lockdownphase (RR 0,74; 95 %-KI [0,70; 0,79]; I^2^ 95,5 %; t^2^ 0,0053; Abb. 2a), die 2. Lockdownphase (RR 0,78; 95 %-KI [0,75; 0,81]; I^2^ 97,1 %; t^2^ 0,0058; Abb. 2b), das Zwischenlockdownintervall (RR 0,88; 95 %-KI [0,86; 0,90]; I^2^ 43,6 %; t^2^ 0; Abb. 2c) und für das gesamte Jahr (RR 0,87; 95 %-KI [0,84; 0,89]; I^2^ 90,4 %; t^2^ 0,0006; Supplement C online; [3, 4, 7–9, 25]).

**Abb. 3:**
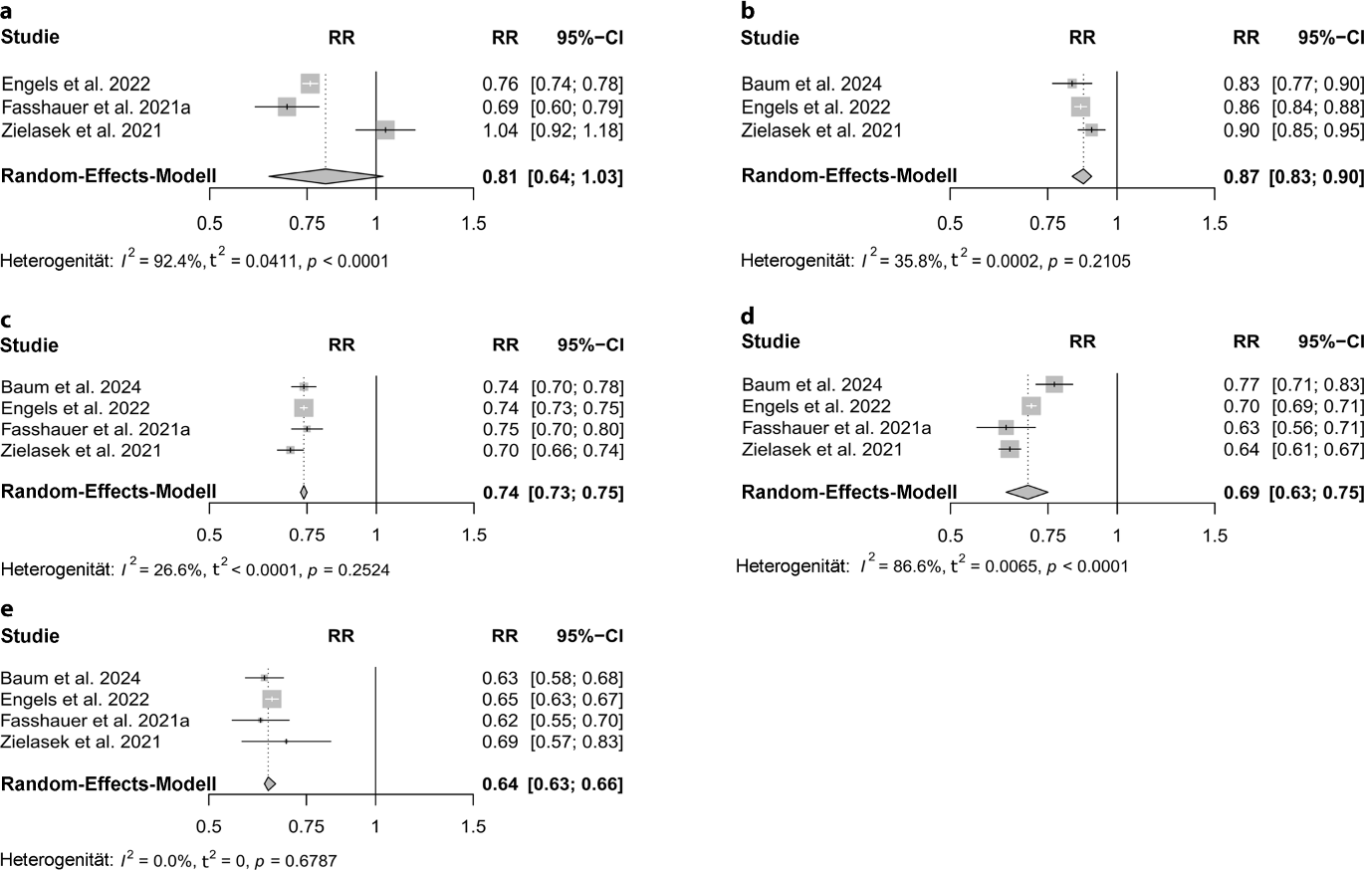
Stationäre Aufnahmen – 1. Lockdownphase. **a **organische psychische Störungen ICD-10 (International Statistical Classification of Diseases and Related Health Problems 10) F0**, b **Substanzkonsumstörungen ICD-10 F1, **c **Schizophrenien, schizotype und wahnhafte Störungen ICD-10 F2, **d** affektive Störungen ICD-10 F3, **e** neurotische, Belastungs- und somatoforme Störungen ICD-10 F4. *RR* Relatives Risiko, *95%-CI* 95 %-Konfidenzintervall

Dabei zeigen sich unterschiedlich ausgeprägte Effekte (und eine geringere Heterogenität) je nach ICD-10-Diagnose-Kategorie. Für die 1. Lockdownphase ergaben sich für die organischen psychischen Störungen (ICD-10 F0): RR 0,81, 95 %-KI [0,64; 1,03], I^2^ 92,4 %; t^2^ 0,0411; für die Substanzkonsumstörungen (F1): RR 0,74, 95 %-KI [0,73; 0,75], I^2^ 26,6 %, t^2^ < 0,0001; für Schizophrenie, schizotype und wahnhafte Störungen (F2): RR 0,87, 95 %-KI [0,83; 0,90], I^2^ 35,8 %, t^2^ 0,0002; für affektive Störungen (F3): RR 0,69, 95 %-KI [0,63; 0,75], I^2^ 86,6 %, t^2^ 0,0065 und für neurotische, Belastungs- und somatoforme Störungen (F4): RR 0,64; 95 %-KI [0,63; 0,66]; I^2^ 0,0 %; t^2^ 0,0 (Tab. e3 online und Abb. 3). Für die Ergebnisse des gesamten Jahres siehe Supplement C online.

### Notaufnahmen

Insgesamt wurden 7 Studien mit mittlerer Qualität (5 Sterne) mit kleiner Beobachtungsebene (C Studien) einbezogen, die die Veränderung der Anzahl der Notaufnahmenvorstellungen während der COVID-19-Pandemie im Vergleich zum Vorjahr untersuchten. Sie zeigten kleine, jedoch signifikante Reduktionen der Gesamtinanspruchnahme wegen psychischer Erkrankungen (Tab. e2 online) in der 1. Lockdownphase (RR 0,87; 95 %-KI [0,79; 0,95]; I^2^ 63,4 %; t^2^ 0,0077) und der 2. Lockdownphase (RR 0,94; 95 %-KI [0,87;1,03]; I^2^ 74,9 %; t^2^ 0,0037; [1, 11, 12, 14, 18, 19, 22]). Für die Details (und einzelne Diagnosekategorien) siehe Supplement D online.

### Ambulante Behandlungen

Zwei Kategorie-A-Studien mit hoher Qualität (6 Sterne) befassten sich mit Maßen der Inanspruchnahme ambulanter psychiatrisch-psychosomatisch-psychotherapeutischer Versorgung. Wegen der geringen Anzahl an Studien und unterschiedlicher Outcomes verzichteten wir auf ein metaanalytisches Modell und stellen hier stattdessen die Ergebnisse der Einzelstudien dar:

Baum et al. [3] berichteten über Reduktionen in der Inzidenz ambulant gestellter Diagnosen während der 1. Lockdownphase (RR 0,90, 95 %-KI [0,89; 0,91]) und der Zwischenlockdownphase (RR 0,94, 95 %-KI [0,93; 0,94]). Während der 2. Lockdownphase gab es keine signifikanten Veränderung (RR 1,0, 95 %-KI [0,99; 1,0]). Mangiapane et al. (RR 0,97, 95 %-KI [0,97; 0,97]; [17]) zeigten für die 1. Lockdownphase Rückgänge der Anzahl an psychotherapeutischen Behandlungsfällen (Einzel- und Gruppentherapien; Tab. e2 online).

### Medikation

Drei Kategorie-A-Studien mit hoher Qualität (6 Sterne) untersuchten die medikamentöse Behandlung in DDD Es ergab sich eine nichtsignifikante Reduktion der verordneten DDD (RR 0,99; 95 %-KI [0,90; 1,07]; I^2^ 100 %; t^2^ 0,0041; [3, 4, 16]; Tab. e2 online und Abb. 2d).

Eine vierte Studie untersuchte die Anzahl der Patienten mit Lithiumverschreibungen bei verschiedenen Diagnosen und wurde daher nicht in die Metaanalyse einbezogen. Hier fand sich eine signifikante Reduktion der Verschreibungen (RR 0,79, 95 %-KI [0,78; 0,90]; [15]).

### Telemedizin

Zur Nutzung von Telemedizin im Bereich ambulanter psychiatrisch-psychotherapeutischer Versorgung fanden sich mit der beschriebenen Suchstrategie keine Studien.

## Diskussion

Der systematische Review und die Metaanalyse zeigten eine signifikante Reduktion der Anzahl stationärer psychiatrisch-psychotherapeutischer Krankenhausaufnahmen im ersten Jahr der COVID-19-Pandemie in Studien mit hoher Qualität und mittlerer bis hoher Beobachtungsebene. Dabei war die Reduktion am ausgeprägtesten während der 1. Lockdownphase, jedoch auch während der 2. Lockdownphase nachweisbar. Auch zwischen den Lockdowns im ersten Jahr waren keine vollständige Erholung oder Nachholeffekte zu beobachten. Es gab eine hohe Heterogenität der Effekte, die sich vermutlich teils durch die unterschiedlich betroffenen ICD-10-Diagnose-Obergruppen erklären lässt: Insbesondere für die 1. Lockdownphase zeigten sich ausgeprägtere Effekte für affektive (ICD-10 F3), neurotische, Belastungs- und somatoforme Störungen (ICD-10 F4) sowie Suchterkrankungen (ICD-10 F1), während diese zum Beispiel für Schizophrenie, schizotype und wahnhafte Störungen (ICD-10 F2) weniger ausgeprägt waren. Für Behandlungen in Notaufnahmen ließ sich eine geringe Reduktion während der 1. Lockdownphase beschreiben, wobei hier die Studien nur aus einzelnen Notaufnahmen stammen, daher nur eine kleine Beobachtungsebene aufweisen und Schlussfolgerungen für Gesamtdeutschland daher kaum möglich sind. Nur zwei Studien zu den Veränderungen der Inanspruchnahme im ambulanten System konnten einbezogen werden, welche über unterschiedliche Indikatoren geringe Veränderungen (u. a. Reduktion der Neudiagnosen) während der 1. Lockdownphase zeigten. Auch bez. des Volumens ambulant verordneter DDD psychotroper Medikation zeigten sich keine signifikanten Veränderungen. Diese Ergebnisse können bez. des Behandlungsvolumens des ambulanten ärztlichen psychiatrisch-psychotherapeutischen Versorgungssystems so interpretiert werden, dass es diesem Versorgungssektor weitgehend gelungen zu sein scheint, sich an die Herausforderungen der Pandemie zu adaptieren. Ähnlich ließ sich auch ein Survey ambulant tätiger Psychiaterinnen und Psychiater interpretieren, der jedoch auf vielfältige Herausforderungen verwies, gerade durch Wegfallen komplementärer (Spezialtherapien, Tagesstätten, Angebote von Wohneinrichtungen, Werkstätten) und rehabilitativer Angebote und Kooperationen [10].

Die signifikanten Reduktionen der Inanspruchnahme im stationären Sektor sind in mehrerlei Hinsicht diskussionswürdig: Keine uns bekannte Studie kann die Frage beantworten, wie sich diese Reduktionen einerseits auf Qualität und Ergebnisse der psychiatrisch-psychotherapeutischen Versorgung und andererseits auf die Effektivität des Infektionsschutzes in stationären Einrichtungen (z. B. operationalisiert über Mortalität oder Post-COVID-Syndrome nach mit stationären Behandlungen in Zusammenhang stehenden SARS-CoV-2-Infektion) ausgewirkt haben. Dass es Auswirkungen auf die Qualität der Versorgung gab, ist wahrscheinlich angesichts des Ausmaßes der Reduktionen, fehlender Hinweise auf Kompensationen im ambulanten System, der systembedingt für den Großteil der Kliniken (jene ohne Regionalbudget oder Einzelleistungsabrechnungsmodell für psychiatrische Institutsambulanzen) hochgradig eingeschränkten Möglichkeiten ambulanter Kompensation, entsprechender Berichte in Surveys [10, 24] sowie insgesamt fehlender regionaler Steuerung der Versorgung.

Dieser Mangel an Wissen und Systemübersicht ist besonders problematisch, da entsprechende Fragen teilweise mit bestehenden versorgungsnahen Daten beantwortet werden könnten. Wie die vorliegende Arbeit zeigt, sind diese jedoch nur mit erheblichem Zeitverzug zugänglich, zudem ist der Zugang für die Wissenschaft insgesamt eingeschränkt und unterliegt Reglementierungen u. a. durch Akteurinnen und Akteure mit Interessenkonflikten. Eine Zusammenführung mit weiteren relevanten Daten, etwa zu Outcomes in der Psychiatrie und Psychotherapie, ist bislang praktisch nicht realisierbar. Strukturen der „pandemic and crisis preparedness“ sollten daher zukünftig so aufgebaut sein, dass sie erlauben diese Fragen zeitnah zu den Ereignissen und gesundheitspolitischen Steuerungsmaßnahmen zu beantworten.

Dieser systematische Review mit Metaanalyse ist mit verschiedenen Limitationen behaftet: Auf methodischer Ebene ist die zeitliche Limitation der Verlaufsbeobachtung anzumerken, da dieser Review ausschließlich das erste Jahr der Pandemie umfasst. Unklar ist der weitere Verlauf im Zuge der Adaption aller Akteurinnen und Akteure an die Situation. Dennoch können hier die unmittelbaren Effekte einer gesamtgesellschaftlich disruptiven Krise abgebildet werden. Die eingeschlossenen Fall-Kontroll-Studien weisen eine hohe Heterogenität auf, sowohl was die Größe der Beobachtungseinheiten als auch der Diagnosegruppen der eingeschlossenen Teilnehmenden betrifft. Die nach Diagnosegruppen geordnete Subgruppenanalyse konnte hier einige Varianz aufklären. Aus inhaltlicher Perspektive gibt es folgende Diskussionspunkte: Erstens wurden nur einige Bestandteile des psychiatrisch-psychotherapeutischen Versorgungssystems untersucht. Nicht Teil der Studie waren z. B. tagesklinische Leistungen, spezifische Leistungen wie z. B. stationäre Therapieeinheiten, ambulante Psychotherapien oder Leistungen psychiatrischer Institutsambulanzen sowie das gesamte komplementäre Versorgungssystem mit ambulanter Pflege, Ergotherapie, Tagesstätten, Werkstätten, Wohneinrichtungen etc., für die es auch Hinweise auf hochgradige Einschränkungen gab. Zweitens sollte leitlinienorientierte Versorgung schwerer psychischer Erkrankungen oft nicht nur aus Einzelinterventionen, sondern ineinandergreifenden Maßnahmen und Versorgungssequenzen bestehen. Wie sich die Effektivität des Versorgungssystems veränderte, diese leitlinienorientierten Kombinationen und Sequenzen zu realisieren, konnte mit vorliegender Methodik nicht untersucht werden – wäre aber ein entscheidendes Qualitätsmerkmal.

Die Stärke dieser Arbeit liegt darin, eine systematische Übersicht zu Veränderungen der Inanspruchnahme im psychiatrisch-psychotherapeutischen Versorgungssystem in Deutschland im ersten Jahr der Krisenlage der COVID-19-Pandemie unter Berücksichtigung der Beobachtungsebene zu liefern. Diese Ergebnisse können als Basis dienen, um aus der Pandemie für eine bessere „crisis preparedness“ zu lernen. Wesentliche offene Forschungsfragen sind: (1) weitere Verläufe der Inanspruchnahme im zweiten Jahr der Pandemie, (2) weitere Untersuchungen auf umfassender Beobachtungsebene zu den Bereichen ambulanter Versorgung, Notaufnahmen, Rehabilitation sowie Eingliederungshilfe und (3) weitere Untersuchungen zur den Folgen der dargestellten Reduktionen.

## Fazit für die Praxis

- Systematischer Review und Metaanalyse zeigen Reduktionen der Inanspruchnahme während der COVID-19(„coronavirus disease 2019“)-Pandemie, insbesondere für das stationäre psychiatrisch-psychotherapeutische Versorgungssystem, hier jedoch je nach Diagnosegruppe in unterschiedlichem Ausmaß.
- Die ambulante Versorgung war weniger eingeschränkt, wenngleich auch weniger Studien vorlagen. Es gab keine Hinweise auf signifikante Veränderungen der Verordnung ambulanter psychotroper Medikation.
- Wesentliche Fragen nach den Auswirkungen der Reduktionen können, wegen in Deutschland fehlender Forschungsdateninfrastruktur, auch mehrere Jahre nach Beginn der Pandemie nicht beantwortet werden.
- Daher sollte eine Surveillance psychiatrisch-psychotherapeutischer Versorgung in zukünftigen Strukturen der „pandemic and crisis preparedness“ abgebildet und auch außerhalb von Krisenzeiten etabliert sein.

## Supplementary Information

Tabellen

Supplement

## Data Availability

Die extrahierten Daten können bei Interesse beim korrespondierenden Autor angefragt werden.
