# Supplementary material for: Utilization of mental health services in Germany during the first year of the COVID-19 pandemic—Systematic review and meta-analysis
Source: Nervenarzt. 2025 Mar 7;96(3):257–65. [Article in German] doi: 10.1007/s00115-025-01812-y (PMC12058919; doi:10.1007/s00115-025-01812-y)
Supplement: Supplementary file 1 — Tabellen [file 115_2025_1812_MOESM1_ESM.pdf]

Studien	Setting	Studienzeiträume					Ergebnismaße	Studienteilnehmer in der Auswertung		Diagnosen (ICD-10)
		vor Pandemie	während Pandemie							
			ganz-jährig	1. Lockdown	zwischen Lockdowns	2. Lockdown		Vor Pandemie	während Pandemie	
Adam et al. 2023	Notaufnahme	09/2019 bis 02/2020	-	-	-	09/2020 bis 02/2021	Anzahl psychiatrischer Vorstellungen (Neudiagnosen) in psychiatrischer Notaufnahme	1.370	1.249	F0, F1, F2, F3, F4, F6
Adorjan et al. 2021	stationär	04/2019 bis 05/2019	-	04/ 2020 bis 05/ 2020	-	-	stationäre und teilstationäre psychiatrische Belegung in 38 Kliniken	6.840	4.218	gesamt
Baum et al. 2024	stationär/ ambulant/ Medikation	01/ 2019 bis 12/2019	+	Stationär: 03/ 2020 bis 05/ 2020; ambulant: 04/2020 bis 06/2020	Stationär: 06/2020 bis 11/2020; ambulant: 07/2020 bis 09/2020	Stationär: 12/2020 bis 02/2021; ambulant: 09/2020 bis 12/ 2020	Anzahl vollstationärer Fälle, ambulant gestellter Diagnosen, Psychopharmaka-Verordnung in DDD – AOKPlus & BKK Daten von KH aus allen Bundesländern	Stationär: 162.905<br>Ambulant: 2.131.186		F10-F45
Engels et al. 2022	stationär/ Medikation	01/2019 bis 12/2019	+	03/2020 bis 05/ 2020	06/2020 bis 11/2020	12/2020	Anzahl der vollstationären Fälle und Psychopharmaka-Verordnung in DDD - AOK Daten von KH aus allen Bundesländern	427.811	368.543	F00-F89
Fasshauer et al. 2021a	stationär	03/2019 bis 05/2019	-	03/2020 bis 05/2020	-	-	Anzahl stationäre Einweisungen wegen psychischer Störungen, Krankenhaus-verweildauer in 67 KH	4.775	3.327	F00-F69
Fasshauer et al. 2021b	stationär	01/2019 bis 12/2019	+	-	-	-	Anzahl der stationären Einweisungen wegen psychischer Störungen, in 13 Krankenhäusern	22.251	19.691	F00-F99 (F0, F1, F2, F3, F4)
Fasshauer et al. 2022	stationär	01/2018 bis 12/2019	+	04/2020 bis 06/2020	07/2020 bis 09/2020	09/2020 bis 12/2020	Anzahl vollstationärer Notfalleinweisungen; Krankenhaus-Verweildauer in „mehreren" psychiatrischen Krankenhäusern	50.943 (mit 2018)<br>25.404 (nur 2019)	22.469	F00-F69
Goldschmidt et al. 2023	Notaufnahme	03/2019 bis 05/2019	-	03/2020 bis 05/2020	-	-	Anzahl Vorstellungen in einer psychiatrischen Notaufnahme	894	813	F0, F1, F2, F3, F4, F6

Goldschmidt et al. 2024	Notaufnahme	09/2019 bis 02/2020	-	03/2020 bis 05/2020	-	09/2020 bis 02/2021	Anzahl Vorstellungen und Einweisungen in psychiatrischer Notaufnahme	1.855	1.900	F0, F1, F2, F3, F4, F6
Kippe et al. 2023	Notaufnahme	03/2019 bis 05/2019 & 09/ 2019 bis 02/2020	-	03/2020 bis 05/2020	-	09/2020 bis 02/2021	Vorstellungen in psychiatrischer Notaufnahme	2.189	1.921	F0, F1, F2, F3, F4, F6
Kriner et al. 2023	Medikation	01/2019 bis 12/2019	+	-	-	-	Lithium-Verschreibungen auf 26 psychiatrischen Stationen	549	437	gesamt
Ludwig et al. 2022	Medikation	01/2019 bis 12/2019	+	-	-	-	Verordnungen von Psychopharmaka Verordnungsvolumen in DDD	-	-	gesamt
Mangiapane et al. 2022	ambulant	01/2019 bis 05/2020	-	01/2020 bis 05/2020	-	-	Anzahl psychotherapeutischer Behandlungsfälle (Einzel- und Gruppentherapien) aus Daten von 16 der 17 Kassenärztlichen Vereinigungen	3.896.892	3.796.335	gesamt
Seifert et al. 2021	Notaufnahme	03/2019 bis 05/2019	-	03/2020 bis 05/2020	-	-	Psychiatrische Notfallvorstellungen in der Notaufnahme eines akademischen Lehrkrankenhauses	476	374	F1, F2, F3, F4, F6
Sobetzko et al. 2021	Notaufnahme	03/2019 bis 05/2019	-	03/2020 bis 05/2020	-	-	Häufigkeit von Vorstellungen und Diagnoseverteilung in Notaufnahme mit psychiatrischem Versorgungsauftrag	374	387	F1, F2, F3, F4, F6
Ullrich et al. 2023	Notaufnahme	03/2019 bis 05/2019	-	03/2020 bis 05/2020	-	-	Anzahl psychiatrischer Notfallvorstellungen in einer Notaufnahme	351	271	gesamt
Zielasek et al. 2021	stationär	03/2019 bis 05/2019	-	03/2020 bis 05/2020	-	-	Anzahl stationärer Aufnahmen in 9 psychiatrischen Krankenhäusern	14.067	10.545	F1, F2, F3, F4, F6

Tab. e1: Die in die Metaanalyse eingeschlossenen 17 Studien.

Setting	Studie	1. Lockdown			Zwischen Lockdowns			2. Lockdown			gesamtes Jahr			Beobachtungs-ebene	mN[illegible]
		RR	95% KI	SF	RR	95% KI	SF	RR	95% KI	SF	RR	95% KI	SF		
Stationär	Adorjan et al. 2021*	0,62	[0,49; 0,78]	0,0748	-	-	-	-	-	-	-	-	-	B	[illegible]
	Baum et al. 2024	0,70	[0,68; 0,72]	0,0097	0,87	[0,85; 0,89]	0,0081	0,73	[0,71; 0,75]	0,0102	0,84	[0,83; 0,85]	0,0055	A	[illegible]
	Engels et al. 2022	0,73	[0,72; 0,74]	0,0034	0,88	[0,87; 0,89]	0,0027	0,83	[0,82; 0,84]	0,0070	0,86	[0,86; 0,86]	0,0019	A	[illegible]
	Fasshauer et al. 2021a	0,70	[0,67; 0,73]	0,0158	-	-	-	-	-	-	-	-	-	B	[illegible]
	Fasshauer et al. 2021b	-	-	-	-	-	-	-	-	-	0,89	[0,87; 0,91]	0,0087	B	[illegible]
	Fasshauer et al. 2022	0,84	[0,81; 0,87]	0,0136	0,90	[0,87; 0,93]	0,0141	0,84	[0,81; 0,87]	0,0139	0,88	0,86; 0,90]	0,0081	B	[illegible]
	Zielasek et al. 2021	0,75	[0,73; 0,77]	0,0097	-	-	-	-	-	-	-	-	-	B	[illegible]
	**Random Effect Model**	**RR 0,74; 95% KI [0,70; 0,79]; I^2 95,5%; t^2 0,0053**			**RR 0,88; 95% KI [0,86; 0,90]; I^2 43,6%; t^2 0**			**RR 0,78; 95% KI [0,75; 0,81]; I^2 97,1%; t^2 0,0058**			**RR 0,87; 95% KI [0,84; 0,89]; I^2 90,4%; t^2 0,0006**				
Notaufnahme	Adam et al. 2023	-	-	-	-	-	-	0,91	[0,84; 0,98]	0,0356	-	-	-	C	[illegible]
	Goldschmidt et al. 2023	0,91	[0,83; 1,00]	0,0441	-	-	-	-	-	-	-	-	-	C	[illegible]
	Goldschmidt et al. 2024	-	-	-	-	-	-	1,02	[0,96; 1,09]	0,0333	-	-	-	C	[illegible]
	Kippe et al. 2023	0,84	[0,75; 0,93]	0,0459	-	-	-	0,90	[0,84; 0,97]	0,0343	-	-	-	C	[illegible]
	Seifert et al. 2021	0,79	[0,69; 0,90]	0,0546	-	-	-	-	-	-	-	-	-	C	[illegible]
	Sobetzko et al. 2021	1,03	[0,89; 1,19]	0,0747	-	-	-	-	-	-	-	-	-	C	[illegible]
	Ulrich et al. 2023	0,77	[0,66; 0,90]	0,0623	-	-	-	-	-	-	-	-	-	C	[illegible]

	**Random Effect Model**	**RR 0,87; 95% KI [0,79; 0,95]; I^2 63,4%; t^2 0,0077**			**-**			**RR 0,94; 95% KI [0,87;1,03]; I^2 74,9%; t^2 0,0037**			**-**				
**Ambulant**	Baum et al. 2024	0,90	[0,89; 0,91]	0,0028	0,94	[0,93; 0,94]	0,0029	1,00	[0,99; 1,0]	0,0031	0,95	[0,94; 0,96]	0.0014	A	[illegible]
	Mangiapane et al. 2022	0,97	[0,97; 0,97]	0,0007	-	-	-	-	-	-	-	-	-	A	[illegible]
**Medikation**	Kriner et al. 2023*	-	-	-	-	-	-	-	-	-	0,79	[0,78; 0,90]	0,0506	B	[illegible]
	Baum et al. 2024	-	-	-	-	-	-	-	-	-	1,02	[0,81; 1,29]	0,1204	A	[illegible]
	Engels et al. 2022	-	-	-	-	-	-	-	-	-	0,93	[0,93; 0,93]	0,0001	A	[illegible]
	Ludwig et al. 2022	-	-	-	-	-	-	-	-	-	1,03	[1,03; 1,03]	0,0001	A	[illegible]
	**Random Effect Model**	**-**			**-**			**-**			**RR 0,99; 95% KI [0,90; 1,07]; I^2 100%; t^2 0,0041**				

* aufgrund des abweichenden Ergebnismaßes wurde die Studie nicht in die Meta-Analyse einbezogen

RR: Relatives Risiko; KI: Konfidenzintervall; SF: Standardfehler; mNOS: modifizierte Newcastle-Ottawa-Skala

Tab. e2: signifikante Verringerung der stationären Inanspruchnahme

Diagnose	Studie	1. Lockdown			Zwischen Lockdowns			2. Lockdown			Das gesamte Jahr			Beobachtungs-ebene	mNOS
		RR	95% KI	SF	RR	95% KI	SF	RR	95% KI	SF	RR	95% KI	SF		
ICD-10 F0	Baum et al. 2024	-	-	-	-	-	-	-	-	-	-	-	-	A	6
	Engels et al. 2022	0,76	[0,74; 0,78]	0,0122	0,90	[0,88; 0,91]	0,0097	0,85	[0,81; 0,90]	0,0236	0,87	[0,86; 0,88]	0,0067	A	6
	Fasshauer et al. 2021a	0,69	[0,60; 0,79]	0,0468	-	-	-	-	-	-	-	-	-	B	6
	Fasshauer et al. 2021b	-	-	-	-	-	-	-	-	-	0,86	[0,81; 0,91]	0,0263	B	6
	Fasshauer et al. 2022	-	-	-	-	-	-	-	-	-	0,85	[0,81; 0,90]	0,0232	B	6
	Zielasek et al. 2021	1,04	[0,92; 1,18]	0,0652	-	-	-	-	-	-	-	-	-	B	6
	**Random Effect Model**	**RR 0,81; 95% KI [0,64; 1,03]; I^2 92,4%; t^2 0,0411**			**-**			**-**			**RR 0,87; 95% KI [0,85; 0,88]; I^2 0,0%; t^2 0,0**				
ICD-10 F1	Baum et al. 2024	0,74	[0,70; 0,78]	0,0190	0,89	[0,86; 0,92]	0,0154	0,73	[0,69; 0,76]	0,0193	0,68	[0,66; 0,70]	0,0084	A	6
	Engels et al. 2022	0,74	[0,73; 0,75]	0,0055	0,85	[0,84; 0,86]	0,0042	0,76	[0,74; 0,78]	0,0102	0,84	[0,83; 0,85]	0,0030	A	6
	Fasshauer et al. 2021a	0,75	[0,70; 0,80]	0,0244	-	-	-	-	-	-	-	-	-	B	6
	Fasshauer et al. 2021b	-	-	-	-	-	-	-	-	-	0,89	[0,86; 0,92]	0,0145	B	6
	Fasshauer et al. 2022	-	-	-	-	-	-	-	-	-	0,89	[0,87; 0,91]	0,0120	B	6
	Zielasek et al. 2021	0,7	[0,66; 0,74]	0,0192	-	-	-	-	-	-	-	-	-	B	6
	**Random Effect Model**	**RR 0,74; 95% KI [0,73; 0,75]; I^2 26,6%;t^2 <0,0001**			**-**			**-**			**RR 0,87; 95% KI [0,84; 0,89]; I^2 99,0%; t^2 0,0162**				

**ICD-10 F2**	Baum et al. 2024	0,83	[0,77; 0,90]	0,0344	0,91	[0,86; 0,97]	0,0270	0,81	[0,74; 0,88]	0,0350	0,90	[0,86; 0,94]	0,0188	A	6
	Engels et al. 2022	0,86	[0,84; 0,88]	0,0111	0,91	[0,90; 0,93]	0,0081	0,97	[0,93; 1,02]	0,0226	0,92	[0,91; 0,93]	0,0058	A	6
	Fasshauer et al. 2021a	-	-	-	-	-	-	-	-	-	-	-	-	B	6
	Fasshauer et al. 2021b	-	-	-	-	-	-	-	-	-	0,92	[0,87; 0,97]	0,0241	B	6
	Fasshauer et al. 2022	-	-	-	-	-	-	-	-	-	0,92	[0,87; 0,97]	0,0259	B	6
	Zielasek et al. 2021	0,90	[0,85; 0,95]	0,0256	-	-	-	-	-	-	-	-	-	B	6
	**Random Effect Model**	**RR 0,87; 95% KI [0,83; 0,90]; I^2 35,8%; t^2 0,0002**			**-**			**-**			**RR 0,92; 95% KI [0,91; 0,93]; I^2 00,0%;t^2 0**				
**ICD-10 F3**	Baum et al. 2024	0,77	[0,71; 0,83]	0,0300	0,94	[0,89; 0,99]	0,0254	0,65	[0,60; 0,70]	0,0263	0,90	[0,87; 0,93]	0,0171	A	6
	Engels et al. 2022	0,70	[0,69; 0,71]	0,0073	0,89	[0,87; 0,90]	0,0062	0,84	[0,81; 0,87]	0,0156	0,86	[0,85; 0,87]	0,0042	A	6
	Fasshauer et al. 2021a	0,63	[0,56; 0,71]	0,0390	-	-	-	-	-	-	-	-	-	B	6
	Fasshauer et al. 2021b	-	-	-	-	-	-	-	-	-	0,87	[0,84; 0,90]	0,0166	B	6
	Fasshauer et al. 2022	-	-	-	-	-	-	-	-	-	0,93	[0,89; 0,97]	0,0223	B	6
	Zielasek et al. 2021	0,64	[0,61; 0,67]	0,0148	-	-	-	-	-	-	-	-	-	B	6
	**Random Effect Model**	**RR 0,69; 95% KI [0,63; 0,75]; I^2 86,6%; t^2 0,0065**			**-**			**-**			**RR 0,89; 95% KI [0,86; 0,91]; I^2 79,9%; t^2 0,0009**				
**ICD-10 F4**	Baum et al. 2024	0,63	[0,58; 0,68]	0,0403	0,86	[0,82; 0,91]	0,0263	0,62	[0,57; 0,67]	0,0412	0,82	[0,79; 0,85]	0,0189	A	6
	Engels et al. 2022	0,65	[0,63; 0,67]	0,0137	0,86	[0,84; 0,88]	0,0089	0,84	[0,80; 0,88]	0,0251	0,84	[0,83; 0,85]	0,0064	A	6

	Fasshauer et al. 2021a	0,62	[0,55; 0,70]	0,0603	-	-	-	-	-	-	-	-	-	B	6
	Fasshauer et al. 2021b	-	-	-	-	-	-	-	-	-	0,85	[0,79; 0,91]	0,0362	B	6
	Fasshauer et al. 2022	-	-	-	-	-	-	-	-	-	0,81	[0,77; 0,85]	0,0244	B	6
	Zielasek et al. 2021	0,69	[0,57; 0,83]	0,0941	-	-	-	-	-	-	-	-	-	B	6
	**Random Effect Model**	**RR 0,64; 95% KI [0,63; 0,66]; I^2 00,0%; t^2 0**			**-**			**-**			**RR 0,84; 95% KI [0,82; 0,84]; I^2 13,6%; t^2 0,0001**				
**ICD-10 F5**	Baum et al. 2024	-	-	-	-	-	-	-	-	-	-	-	-	A	6
	Engels et al. 2022	0,68	[0,62; 0,76]	0,0343	0,98	[0,92; 1,05]	0,0326	0,97	[0,81; 1,16]	0,0882	0,90	[0,86; 0,95]	0,0215	A	6
	Fasshauer et al. 2021a	-	-	-	-	-	-	-	-	-	-	-	-	B	6
	Fasshauer et al. 2021b	-	-	-	-	-	-	-	-	-	-	-	-	B	6
	Fasshauer et al. 2022	-	-	-	-	-	-	-	-	-	1,01	[0,81; 1,26]	0,1158	B	6
	Zielasek et al. 2021	-	-	-	-	-	-	-	-	-	-	-	-	B	6
	**Random Effect Model**	**-**			**-**			**-**			**-**				
**ICD-10 F6**	Baum et al. 2024	-	-	-	-	-	-	-	-	-	-	-	-	A	6
	Engels et al. 2022	0,73	[0,70; 0,77]	0,0184	0,92	[0,89; 0,95]	0,0153	0,85	[0,78; 0,93]	0,0390	0,89	[0,87; 0,91]	0,0107	A	6
	Fasshauer et al. 2021a	-	-	-	-	-	-	-	-	-	-	-	-	B	6
	Fasshauer et al. 2021b	-	-	-	-	-	-	-	-	-	-	-	-	B	6

	Fasshauer et al. 2022	-	-	-	-	-	-	-	-	-	0,96	[0,86; 1,07]	0,0533	B	6
	Zielasek et al. 2021	0,73	[0,63; 0,85]	0,0564	-	-	-	-	-	-	-	-	-	B	6
	**Random Effect Model**	**-**			**-**			**-**			**-**				

RR: Relatives Risiko; KI: Konfidenzintervall; SF: Standardfehler; mNOS: modifizierte Newcastle-Ottawa-Skala

ICD-10 F0: Organische, einschließlich symptomatischer psychischer Störungen; ICD-10 F1: Psychische und Verhaltensstörungen durch psychotrope Substanzen; ICD-10 F2: Schizophrenie, schizotype und wahnhafte Störungen; ICD-10 F3: Affektive Störungen; ICD-10 F4: Neurotische, Belastungs- und somatoforme Störungen; ICD-10 F5: Verhaltensauffälligkeiten mit körperlichen Störungen und Faktoren; ICD-10 F6: Persönlichkeits- und Verhaltensstörungen

Tab. e3: unterschiedlich ausgeprägte Effekte (und eine geringere Heterogenität) je nach ICD-10-Diagnose-Kategorie
