# Supplementary material for: Utilization of mental health services in Germany during the first year of the COVID-19 pandemic—Systematic review and meta-analysis
Source: Nervenarzt. 2025 Mar 7;96(3):257–65. [Article in German] doi: 10.1007/s00115-025-01812-y (PMC12058919; doi:10.1007/s00115-025-01812-y)

Supplement A - Suchstrategie

Effects of the COVID-19 pandemic on the utilization of mental health services in Germany

Search strategies

Search strategy PubMed

1."mental illness"[Title/Abstract]

2."mental health"[MeSH Terms]

3."psychiatric emergency admission*"[Title/Abstract]

4."psychiatric hospitalization*"[Title/Abstract]

5."inpatient admission*"[Title/Abstract]

6."increased admission*"[Title/Abstract]

7."inpatient psychiatry"[Title/Abstract]

8."mental healthcare"[Title/Abstract]

9."self harm"[Title/Abstract]

10."substance use"[Title/Abstract]

11."outpatient psychiatr*"[Title/Abstract]

12."psychiatric presentation*"[Title/Abstract])

13."emergency presentation*"[Title/Abstract]

14."suicidal behavior"[Title/Abstract])

15. OR/#1-#14

16."psychiatric emergency department"[Title/Abstract]

17."psychiatric admission*"[Title/Abstract]

18."multicenter study"[Title/Abstract]

19."mental health cohort"[Title/Abstract]

20."cross sectional study"[Title/Abstract]

21."interrupted time series"[Title/Abstract]

22."retrospective study"[Title/Abstract]

23."retrospective cohort"[Title/Abstract]

24."electronic health record study"[Title/Abstract]

25."service database"[Title/Abstract]

26."mental healthcare data"[Title/Abstract]

27."routine data"[Title/Abstract]

28."european psychiatric association"[Title/Abstract]

29."psychiatric emergency service"[Title/Abstract]

30."primary care electronic health records"[Title/Abstract]

31."psychosis clinic"[Title/Abstract])

32. OR/#16-#31

33."coronavirus"[Title/Abstract]

34."corona"[Title/Abstract]

35."covid 19"[Title/Abstract]

36."2019 ncov"[Title/Abstract]

37."sars cov 2"[Title/Abstract]

38."covid 19"[MeSH Terms]

39."sars cov 2"[MeSH Terms]

40. OR/#33-#39

41. #15 AND #32 AND #40

42. "germany"[Title/Abstract]

43. "german*"[Title/Abstract]

44. "germany"[MeSH Terms]

45. "german*"[MeSH Terms]

46. OR/#42-#45

47. #41 AND #46

48. Filter 2022-2023

Ovid PsycINFO

S1 TI (coronavirus) OR AB (coronavirus)

S2 TI (corona) OR AB (corona)

S3 TI (covid-19) OR AB (covid-19)

S4 TI (sars-cov-2) OR AB (sars-cov-2)

S5 MA (covid-19)

S6 MA (sars-cov-2)

S7 S1 OR S2 OR S3 OR S4 OR S5 OR S6

S8 TI (prescri* N3 (mental N1 health)) OR AB (prescri* N3 (mental N1 health))

S9 TI (utilization N2 (mental N1 health)) OR AB (utilization N2 (mental N1 health))

S10 TI (increas* N3 (mental N1 health)) OR AB (increas* N3 (mental N1 health))

S11 TI (decreas* N3 (mental N1 health)) OR AB (decreas* N3 (mental N1 health))

S12 TI (emergenc* N3 (mental N1 health)) OR AB (emergenc* N3 (mental N1 health))

S13 TI (emergenc* N3 (presentation)) OR AB (emergenc* N3 (presentation))

S14 TI (inpatient* N3 (mental N1 health)) OR AB (inpatient* N3 (mental N1 health))

S15 TI (outpatient* N3 (mental N1 health)) OR AB (outpatient* N3 (mental N1 health))

S16 TI (hospitalization N3 (mental N1 health)) OR AB (hospitalization N3 (mental N1 health))

S17 TI psychiatr* OR AB psychiatr*

S18 TI ((substance N1 use) or (substance N1 abuse)) OR AB ((substance N1 use) or (substance N1 abuse))

S19 MA (mental health)

S20 TI (mental N1 health) OR AB (mental N1 health)

S21 S8 OR S9 OR S10 OR S11 OR S12 OR S13 OR S14 OR S15 OR S16 OR S17 OR S18 OR S19 OR S20

S22 TI (longitudinal N2 stud*) OR AB (longitudinal N2 stud*)

S23 TI (prospective N1 stud*) OR AB (prospective N1 stud*)

S24 TI (retrospective N1 stud*) OR AB (retrospective N1 stud*)

S25 TI (quantitative N1 stud*) OR AB (quantitative N1 stud*)

S26 TI (data N1 resource) OR AB (data N1 resource)

S27 TI (electronic health records) OR AB (electronic health records)

S28 TI (psychiatric admission*) OR AB (psychiatric admission*)

S29 S22 OR S23 OR S24 OR S25 OR S26 OR S27 OR S28

S30 S7 AND S21 AND S29

S31 TI german* OR AB german*

S32 MA german*

S33 S31 OR S32

S34 S30 AND S33

Filter 2022-2023

Embase

#1 coronavirus:ab,ti,kw

#2 corona:ab,ti,kw

#3 'covid 19':ab,ti,kw

#4 'sars cov 2':ab,ti,kw

#5 'coronavirus disease 2019'/exp

#6 'sars coronavirus'/exp

#7 (prospective NEAR/2 stud*):ab,ti,kw

#8 (retrospective NEAR/2 stud*):ab,ti,kw

#9 (quantitative NEAR/2 stud*):ab,ti,kw

#10 longitudinal:ab,ti,kw

#11 'electronic health records':ab,ti,kw

#12 (utilisation NEAR/2 'mental health'):ab,ti,kw

#13 (utilization NEAR/2 'mental health'):ab,ti,kw

#14 (increas* NEAR/3 'mental health'):ab,ti,kw

#15 (decreas* NEAR/3 'mental health'):ab,ti,kw

#16 (emergenc* NEAR/3 'mental health'):ab,ti,kw

#17 (inpatient* NEAR/2 'mental health'):ab,ti,kw

#18 (outpatient* NEAR/2 'mental health'):ab,ti,kw

#19 (hospitalization NEAR/3 'mental health'):ab,ti,kw

#20 'mental health'/exp

#21 (emergenc* NEAR/1 presentation):ab,ti,kw

#22 (prescri* NEAR/3 'mental health'):ab,ti,kw

#23 (psychiatr* NEAR/3 'mental health'):ab,ti,kw

#24 (hospitalisation NEAR/3 'mental health'):ab,ti,kw

#25 #7 OR #8 OR #9 OR #10 OR #11

#26 #1 OR #2 OR #3 OR #4 OR #5 OR #6
#27 #12 OR #13 OR #14 OR #15 OR #16 OR #17 OR #18 OR #19 OR #20 OR #21 OR #22 OR #23 OR #24

#28 #25 AND #26 AND #27

#29 #28 AND [01-06-2022]/sd

#30 'germany'/exp OR german*:ti,ab

#31 #29 AND #30

SUPPLEMENT	B	mNOS										
Studie	cohort<br>1) truly representative of the review's target group* ★<br>2) somewhat representative of the reviews target group ★<br>3) selected group of users eg patients frequenting one institution/emergency department /<br>4) no description of the derivation of the cohort /<br>Response options:<br><br>*patients with pre-existing or newly diagnosed mental disorders (ICD-10, DSM-5), aged 18 years or older (i.e. adult mental healthcare system)	selected number and notes1), 2), 3	**2. Selection of the non-exposed cohort**<br><br>1) drawn from the same community/basic population as the exposed cohort (e.g. other time interval but same region/structure of population, groups have same eligibility criteria, the only difference is exposure/non-exposure/point of assessment) ★<br>2) drawn from a different source /<br>3) no description of the derivation of the non exposed cohort /	selected number and notes	**4. Demonstration that outcome of interest was not confounded from start of study**<br><br>1) yes* ★<br>2) no /<br><br>*demonstrated if eligibility criteria do not differ except for observation periods	selected number and notes	**5. Comparability of participants on the basis of the design or analysis***<br><br>1) study controls for exposure to Covid-pandemic ★<br>2) study controls for any additional factor: exposure to any other epidemic or pandemic infectious disease outbreaks as well as other macro stressors like natural or human-made disasters<br><br>*A maximum of two stars can be given to Compatibility	selected number and notes	**6. Assessment of outcome**<br><br>1) classified quantitative data (e.g, register or routine data) ★<br>2) ad-hoc surveys /<br>3) no description /	selected number and notes	**7. Does the observation period include a local lockdown or high frequency phase?**<br>1) yes ★<br>2) no /	selected number and notes
Adorjan 2021	☆	2)Aufgrund der geringen Anzahl der teilnehmenden Kliniken ist die Aussagekraft der Studie auf einen Querschnitt der deutschlandweit insgesamt 38 untersuchten Kliniken begrenzt.	☆	1) keine Änderung der Einschlusskriterien	☆	same time period Vergleich zum Vorjahr	☆	1)Um die strukturelle Leistungsfähigkeit psychiatrischer Kliniken in Deutschland während der COVID(„coronavirus disease")-19-Pandemie beurteilen zu können, wurde im März 2020 eine Umfrage durchgeführt, in der die Corona-bedingten Veränderungen der Versorgungsstrukturen während der ersten Welle der Pandemie erfasst wurden.	☆	1)Der Fragebogen wurde an den Universitätsklinika München (LMU) und Mainz konzipiert. Die Durchführung der Befragung und die Datensammlung erfolgte über die Geschäftsstelle der DGPPN in Berlin. Es wurden keine personenbezogenen Daten erhoben. Die ethischen Aspekte einer anonymisierten Institutsbefragung wurden von der Ethikkommission der LMU geprüft, ein Ethikvotum liegt vor.	☆	1) Der Erfassungszeitraum betrug einen Monat, von Mitte April bis Mitte Mai 2020.
Engels 2022	☆	1)Im Rahmen des aktuellen Psychiatrie-Barometers des Deutschen Krankenhausinstituts (DKI) belegen die Daten einer jährlich durchgeführten Repräsentativerhebung in insgesamt 312 psychiatrischen und psychosomatischen Einrichtungen starke Rückgänge der Auslastung in der teil- und vollstationären Psychiatrie während der ersten Hochphase der Pandemie von März bis Juni 2020	☆	1) keine Änderung der Einschlusskriterien	☆	same time period Vergleich zum Vorjahr	☆	1)Im vorliegenden Beitrag wird die veränderte Inanspruchnahme des psychiatrischen Versorgungsangebots während der Covid-19-Pandemie im Jahr 2020 im Vergleich zum Vorjahr analysiert.	☆	1)Es wurden anonymisierte GKV-Routinedaten des Wissenschaftlichen Instituts der AOK (WIdO) analysiert.	☆	1)Betrachtet werden die Entwicklung der Krankenhausfälle im Rahmen von voll- und teilstationären Versorgungsleistungen sowie die Auslastung in Hochschulambulanzen und psychiatrischen Institutsambulanzen während der Lockdown-Maßnahmen über verschiedene Diagnosegruppen hinweg.
Fasshauer 2021a	☆	2) To address these open points, data from 67 hospitals of a large and regionally diverse German health care provider ("Helios hospitals") were evaluated concerning number of admissions for psychiatric disorders and length of hospital stay.	☆	1) keine Änderung der Einschlusskriterien	☆	same time period Vergleich zum Vorjahr	☆	1) A retrospective study was performed analyzing claims data from a large German Hospital network during the COVID-19 outbreak (study period: March 13–May 21, 2020) as compared to periods directly before the outbreak (same year control: January 1–March 12, 2020) and one year earlier (previous year control: March 13–May 21, 2019)	☆	1) Due to the retrospective study of anonymized data, informed consent was not obtained.	☆	1) Psychiatric emergency hospital admissions for distinct psychiatric disorders and length of inpatient stay in the hospital during the Coronavirus disease 2019 (COVID-19) outbreak
Fasshauer 2021b	☆	2) To address this open point, data from 13 hospitals of a large and regionally diverse German health care provider (HELIOS hospitals) comprising a total of 64,502 admissions for psychiatric diagnoses between 2018 and 2020 were examined.	☆	1) keine Änderung der Einschlusskriterien	☆	same time period Vergleich zum Vorjahr	☆	1) no study so far has elucidated the impact ofthe COVID-19 pandemic year 2020 on urgent and involuntary admissions, as well as coercive measures, compared to previous non-COVID-19 years.	☆	1) Due to the retrospective study of anonymized data, informed consent was not obtained.	☆	1)Three periods were defined to compare inpatient admissions for selected psychiatric diagnosis groups. The study period (January 1, 2020 to December 31, 2020) was compared to two control periods, i.e., January 1, 2019 to December 31, 2019 and January 1, 2018 to December 31, 2018.
Fasshauer 2022	☆	2) Die Größe der Stichprobenerhebung sowie die regionale Verteilung der Krankenhäuser über alle Bundesländer stellen Stärken der Arbeit dar. Zudem liegen für jede untersuchte Diagnosegruppe repräsentative Daten vor	☆	1) keine Änderung der Einschlusskriterien	☆	same time period Vergleich zum Vorjahr	☆	1) Die Auswirkungen der COVID-19-Pandemie in 2020 auf die Anzahl der täglichen psychiatrischen Notfallaufnahmen und die stationäre psychiatrische Aufenthaltsdauer wurde mit Vorjahren verglichen	☆	1) Administrative Daten wurden von QlikView (QlikTech, Radnor, Pennsylvania, USA) ausgelesen und die Daten pseudonymisiert gespeichert. Da es sich um eine retrospektive Studie mit anonymisierten Daten handelt, ist eine Einwilligung zur Verarbeitung der Daten nicht notwendig	☆	1) Das ganze Jahr mit zwei Lockdown Phasen ist erfasst
Goldschmidt 2023	/	3) that this study is limited to one site, where data was collected	☆	1) keine Änderung der Einschlusskriterien	☆	same time period Vergleich zum Vorjahr	☆	1) Retrospective chart review of all pED presentations and admissions during the frst wave of Covid-19 pandemic in Germany	☆	1) We conducted a retrospective chart review of clinical documentation records of all presentations at the psychiatric department at St. Hedwig Hospital (SHK) in Berlin	☆	1) Retrospective chart review of all pED presentations and admissions during the frst wave of Covid-19 pandemic in Germany
Kippe 2023	/	3) our data is limited to one study site and may therefore be infuenced by specifc circumstances that limit generalizability	☆	1) keine Änderung der Einschlusskriterien	☆	same time period Vergleich zum Vorjahr	☆	1) We conducted a retrospective analysis of clinical records of all patients attending the psychiatric clinic of Charité University Berlin at St. Hedwig Hospital (SHK) in Berlin during the frst (3/2/2020 – 5/24/2020 "frst-wave") and the second (9/15/2020 – 3/1/2021 "second-wave") wave of the Covid19 pandemic in Germany.	☆	1) We conducted a retrospective analysis of clinical records of all patients attending the psychiatric clinic of Charité University Berlin at St. Hedwig Hospital (SHK) in Berlin during the frst (3/2/2020 – 5/24/2020 "frst-wave") and the second (9/15/2020 – 3/1/2021 "second-wave") wave of the Covid19 pandemic in Germany.	☆	1) We conducted a retrospective analysis of clinical records of all patients attending the psychiatric clinic of Charité University Berlin at St. Hedwig Hospital (SHK) in Berlin during the frst (3/2/2020 – 5/24/2020 "frst-wave") and the second (9/15/2020 – 3/1/2021 "second-wave") wave of the Covid19 pandemic in Germany.
Ludwig 2022	☆	1) Arzneimittelreport 2021	☆	1) keine Änderung der Einschlusskriterien	☆	same time period Vergleich zum Vorjahr	☆	1) Arzneimiitelreport während des Pandemiejahrs	☆	1) Die Analysen im Arzneiverordnungs-Report 2022 basieren auf den Verordnungsdaten des GKV Arzneimittelindex für ambulante Patienten, der in der Trägerschaft des AOK Bundesverbandes vom Wissenschaftlichen Institut der AOK (WIdO) erstellt wurde	☆	1) 2021 zweites Lockdown in DE
Mangiapane et al. 2022	☆	1) Veränderung der vertragsärztlichen Leistungsinanspruchnahme während der COVID-Krise Tabellarischer Trendreport bis zum 1. Halbjahr 2022	☆	1) keine Änderung der Einschlusskriterien	☆	same time period Vergleich zum Vorjahr	☆	1) Veränderung der vertragsärztlichen Leistungsinanspruchnahme während der COVID-Krise Tabellarischer Trendreport bis zum 1. Halbjahr 2022	☆	1) Für den vorliegenden Trendreport wurden dem Zi von 16 der 17 Kassenärztlichen Vereinigungen (KVen, ohne Mecklenburg-Vorpommern) aggregierte Informationen aus den Abrechnungsdaten des Zeitraumes 1. Quartal 2019 bis 2. Quartal 2022 übermittelt.	☆	1) Veränderung der vertragsärztlichen Leistungsinanspruchnahme während der COVID-Krise Tabellarischer Trendreport bis zum 1. Halbjahr 2022
Seifert 2021	/	3) his study aims to present the immediate efects of the pandemic on patients presenting in the psychiatric emergency department (PED) of Hannover Medical School	☆	1) keine Änderung der Einschlusskriterien	☆	same time period Vergleich zum Vorjahr	☆	1) his study aims to present the immediate efects of the pandemic on patients presenting in the psychiatric emergency department (PED) of Hannover Medical School	☆	1) All data was de-identifed by pseudonymization	☆	1) March 16th, 2020 was selected as starting point as this was the day that marked the closing of all schools, stores selling non-essential goods, and recreational facilities in the state of Lower Saxony, Germany.
Sobetzko 2021	/	3) Sie stellen die Entwicklung in einer großstädtischen interdisziplinären Notaufnahme dar und sind nicht repräsentativ für andere Regionen oder ländliche Gebiete.	☆	1) keine Änderung der Einschlusskriterien	☆	same time period Vergleich zum Vorjahr	☆	1) Es wurden psychiatrische Notfallkontakte in der interdisziplinären Notfallaufnahme der Evang. Kliniken Essen-Mitte für den Zeitraum des Lockdowns vom 15.03.2020 bis zum 04.05.2020 mit den Notfällen im gleichen Zeitraum 2019 verglichen.	☆	1) Die Daten der Patienten wurden retrospektiv erhoben. Die Daten wurden mit Hilfe der Aufnahmedokumentation, der Notfalldokumentation, SAP und iMedOne erfasst und analysiert	☆	1) Es wurden psychiatrische Notfallkontakte in der interdisziplinären Notfallaufnahme der Evang. Kliniken Essen-Mitte für den Zeitraum des Lockdowns vom 15.03.2020 bis zum 04.05.2020 mit den Notfällen im gleichen Zeitraum 2019 verglichen.
Zielasek 2021	☆	2)Our analysis is limited by the regional constraint. However, with more than four million inhabitants served by the LVR Clinics Association, we provide the first analysis of the full data of one of the largest providers of psychiatric inpatient and outpatient hospital care in Germany.	☆	1) keine Änderung der Einschlusskriterien	☆	same time period Vergleich zum Vorjahr	☆	1)Objectives: We conducted an analysis of the changes of inpatient and day patient mental health care utilization in an association of psychiatric hospitals during the COVID-19 pandemic.	☆	1)We analyzed the routine data of all inpatient and day-patient admission cases in all departments of the nine psychiatric hospitals of the LVR (general psychiatry, child and adolescent psychiatry, psychosomatic medicine, geriatric psychiatry, and addiction psychiatry) during the reporting periods from March 18 until May 31 for the years 2019 and 2020.	☆	1)Until the end of the observation period, which included the end of the first wave of COVID-19 in Germany, admission rates remained lower than in the corresponding time period of the previous year.
Baum 2024	☆	1) This study utilizes a large set of claims data covering a total of 8.8 million insured individuals across Germany, however, with a bias towards the region of Saxony. Nonetheless, since the majority of the results were also confirmed in the subset of the BKK data that is representative for Germany [40] (see Supplement III), the results can be generalized to the whole of Germany.	☆	1) keine Änderung der Einschlusskriterien	☆	same time period Vergleich zum Vorjahr	☆	1) We analyzed changes in inpatient and outpatient mental health service utilization over the course of the first two lockdown phases (LDPs) of the pandemic in 2020 compared to a pre-COVID-19 reference period dating from March 2019 to February 2020 using a time series forecast model.	☆	1) Therefore, the goal of this study was to comprehensively and systematically analyze in a large routine data set, which sectors (inpatient services and outpatient psychiatric, psychopharmacotherapeutic and psychotherapeutic services) and which patient populations stratified according to predefined diagnostic groups of mental disorders were affected by reductions in mental health service utilization in Germany during the first year of the COVID-19 pandemic	☆	1) We analyzed changes in inpatient and outpatient mental health service utilization over the course of the first two lockdown phases (LDPs) of the pandemic in 2020 compared to a pre-COVID-19 reference period dating from March 2019 to February 2020 using a time series forecast model.
Engels 2024	☆	1) In this retrospective cohort study, we analyzed health insurance claims data of the "Wissenschaftliches Institut der AOK" ("WIdO") for the period from January 1, 2018, to February 28, 2021. WIdO is the scientific institute of the AOK, which is the largest association of statutory health insurance companies in Germany. In total, the eleven autonomous companies of the AOK cover 26.8 million insurants (reference year 2019). This corresponds to about one third of the German population.	☆	1) keine Änderung der Einschlusskriterien	☆	same time period Vergleich zum Vorjahr	☆	1) Purpose Major lockdowns were imposed in Germany from March until May 2020 and from December 2020 until May 2021. We studied the influence of these lockdowns, the strain on intensive care units and the strictness of COVID-19-related containment strategies on the utilization of mental health care among patients with severe mental disorders	☆	1) In this retrospective cohort study, we analyzed health insurance claims data of the "Wissenschaftliches Institut der AOK" ("WIdO") for the period from January 1, 2018, to February 28, 2021. WIdO is the scientific institute of the AOK, which is the largest association of statutory health insurance companies in Germany. In total, the eleven autonomous companies of the AOK cover 26.8 million insurants (reference year 2019). This corresponds to about one third of the German population.	☆	1) Purpose Major lockdowns were imposed in Germany from March until May 2020 and from December 2020 until May 2021. We studied the influence of these lockdowns, the strain on intensive care units and the strictness of COVID-19-related containment strategies on the utilization of mental health care among patients with severe mental disorders
Adam 2023	/	3)This study focuses on the number of psychiatric new-onset diagnoses in a psychiatric emergency department (pED) in Berlin, Germany during the second wave of the pandemic	☆	1) keine Änderung der Einschlusskriterien	☆	same time period Vergleich zum Vorjahr	☆	1)This study focuses on the number of psychiatric new-onset diagnoses in a psychiatric emergency department (pED) in Berlin, Germany during the second wave of the pandemic	☆	1) Clinical routine chart data documented in ORBIS®, the digital hospital software, from all patients from both time periods was extracted, including the pED cases and the cases with inpatient treatment.	☆	1)This study focuses on the number of psychiatric new-onset diagnoses in a psychiatric emergency department (pED) in Berlin, Germany during the second wave of the pandemic
Ullrich 2023	/	3) Die Ergebnisse dieser Untersuchung sind nicht repräsentativ. Aufgrund der lokalen rettungsdienstlichen Gegebenheiten wird mit hoher Wahrscheinlichkeit ein Großteil aller psychiatrischen Patienten in Siegen erfasst.	☆	1) keine Änderung der Einschlusskriterien	☆	same time period Vergleich zum Vorjahr	☆	1) Das Ziel dieser Arbeit war die Identifizierung von Auswirkungen während des durchgeführten Lockdowns auf psychiatrische Notfallvorstellungen.	☆	1) Die Datenerhebung erfolgte durch einen Facharzt für Psychiatrie und Psychotherapie (H.K.) mit langjähriger Berufserfahrung im Bereich der Akut- und Notfallpsychiatrie. Hierbei konnte auf vollständig digitalisierte Patientenakten zurückgegriffen werden. Auch die handschriftlichen Dokumentationen wie z.B. Notarztprotokolle etc. wurden standardisiert zeitnah eingescannt und lagen somit in digitaler Form dem Studienarzt vor.	☆	1) Das Ziel dieser Arbeit war die Identifizierung von Auswirkungen während des durchgeführten Lockdowns auf psychiatrische Notfallvorstellungen.
Goldschmidt 2024	/	3) A further limitation is that we only gathered information about patients BIBP in a single-center psychiatric emergency department. Extrapolation of results should therefore be done with caution.	☆	1) keine Änderung der Einschlusskriterien	☆	same time period Vergleich zum Vorjahr	☆	1) The primary goal of this exploratory study is to identify predictive factors for PES presentations BIBP during the COVID-19 pandemic.	☆	1) A retrospective chart review of case documentation records of presentations to the PES of Charité Berlin at St. Hedwig Hospital (SHK) was conducted during the first and second wave of the COVID-19 pandemic in Germany in this monocentric study.	☆	1) A retrospective chart review of case documentation records of presentations to the PES of Charité Berlin at St. Hedwig Hospital (SHK) was conducted during the first and second wave of the COVID-19 pandemic in Germany in this monocentric study.
Kriner 2023	☆	2) Up to 26 psychiatric hospitals in Bavaria are participating in the survey.	☆	1) keine Änderung der Einschlusskriterien	☆	same time period Vergleich zum Vorjahr	☆	1) Daten über das ganze Jahr	☆	1) Since 2014, the ongoing Pharmako-EpiVig project collects data on two reference days a year in a cross-sectional approach. Up to 26 psychiatric hospitals in Bavaria are participating in the survey. A list with all participating hospitals can be found in Additional file 1. All inpatients being treated at the participating hospitals on the reference days are included in the survey (Pharmako-Epidemiologie und -Vigilanz 2023). Patients' year of birth, sex, ICD-10-codes of main conditions treated during the hospital stay, i.e. principal psychiatric diagnoses, and of secondary psychiatric as well as somatic diagnoses, along with all drugs administered that day, including daily dosages are reported. In addition, sADRs that occurred within two weeks before the reference day are reported. The questionnaire can be found in Additional file 1. To improve data quality, BIDAQ performs return control and data cleansing procedures. Furthermore, BIDAQ also evaluates drug–drug interactions of prescribed medication using the internet-based drug–drug interaction program mediQ (mediQ-Interaktionsdatenbank 2023). The dataset analyzed for this study includes inpatients from departments of general psychiatry, addiction medicine, geriatric psychiatry, psychosomatic medicine and affiliated departments in participating Bavarian district hospitals from 2014 to 2021. The study protocol and analysis has been approved by the ethics review committee of the Technical University Munich (TUM).	☆	1) Daten über das ganze Jahr

Supplement C –Aufnahmen stationär - Jahr

a Aufnahmen stationär –Jahr –alle Diagnosen

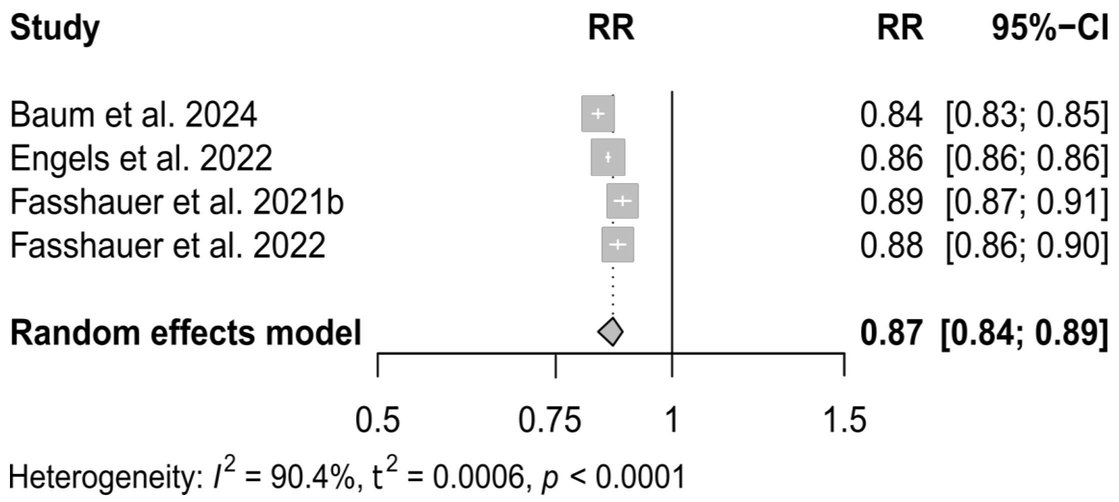

b Aufnahmen stationär –Jahr –F0

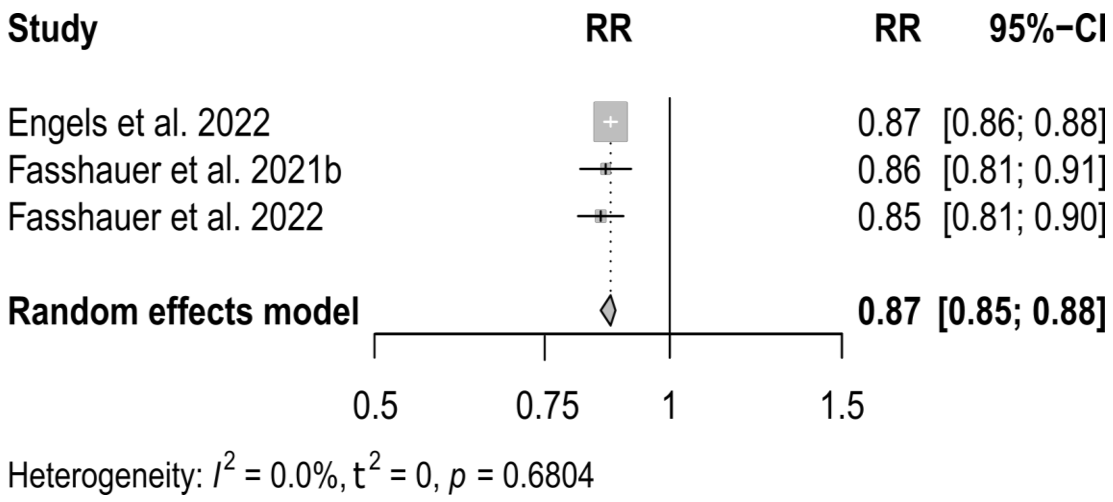

c Aufnahmen stationär –Jahr –F1

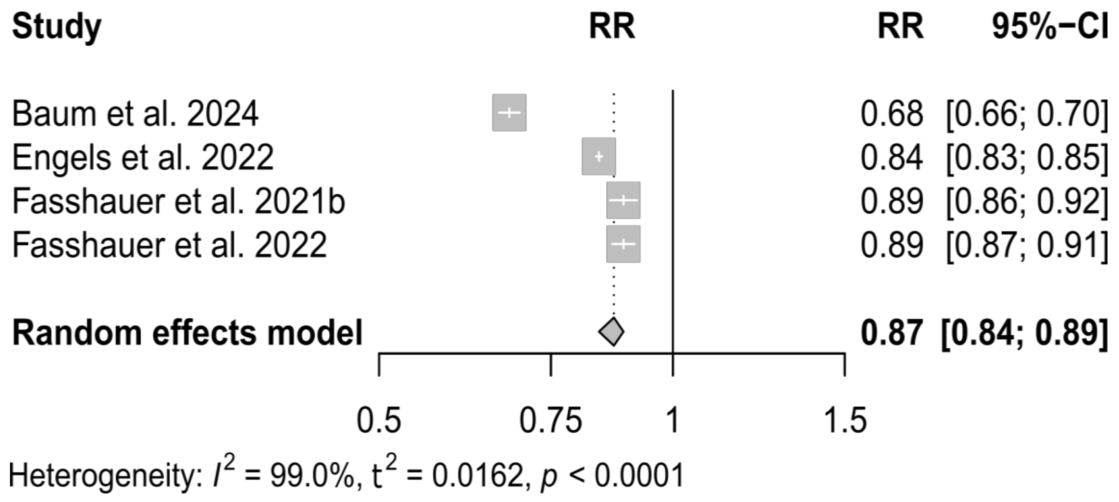

d Aufnahmen stationär –Jahr –F2

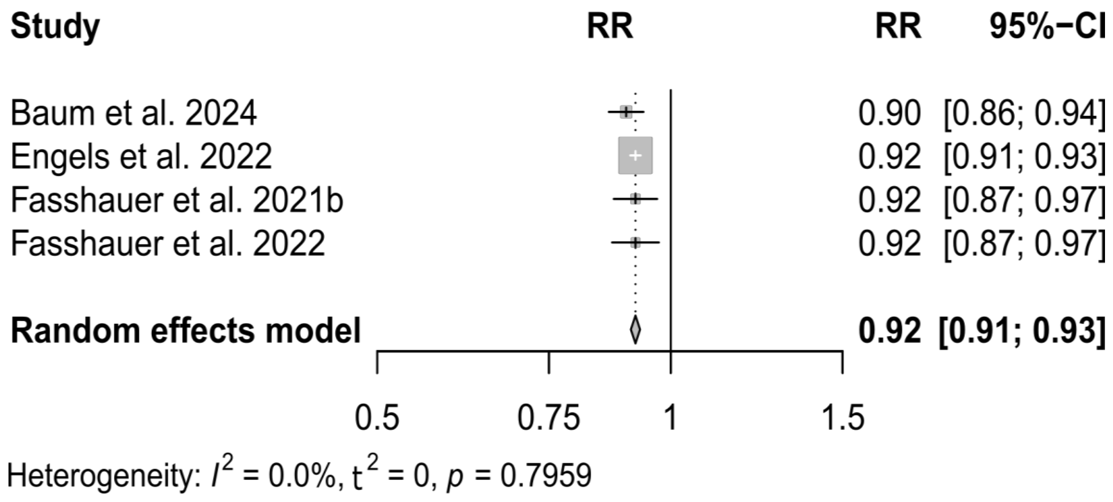

e Aufnahmen stationär –Jahr –F3

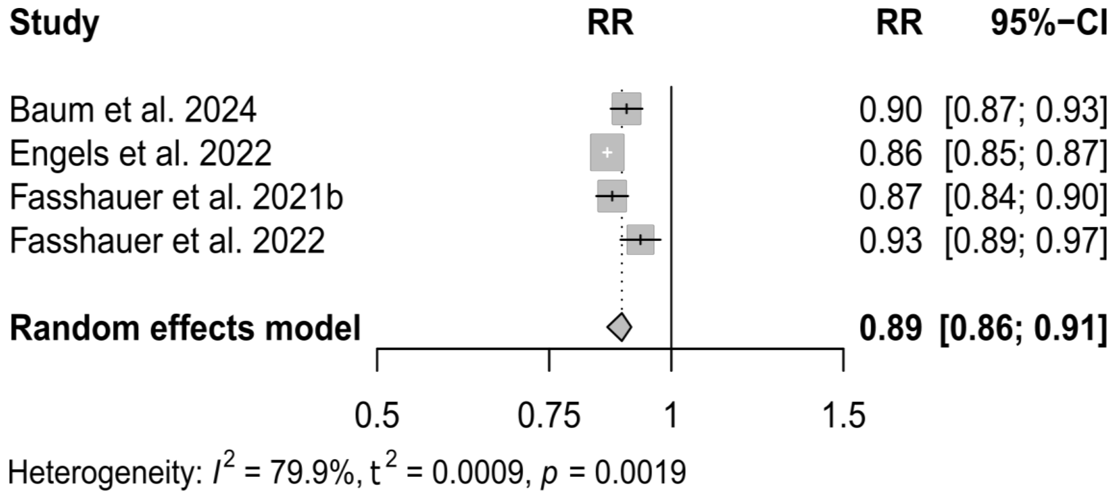

f Aufnahmen stationär –Jahr –F4

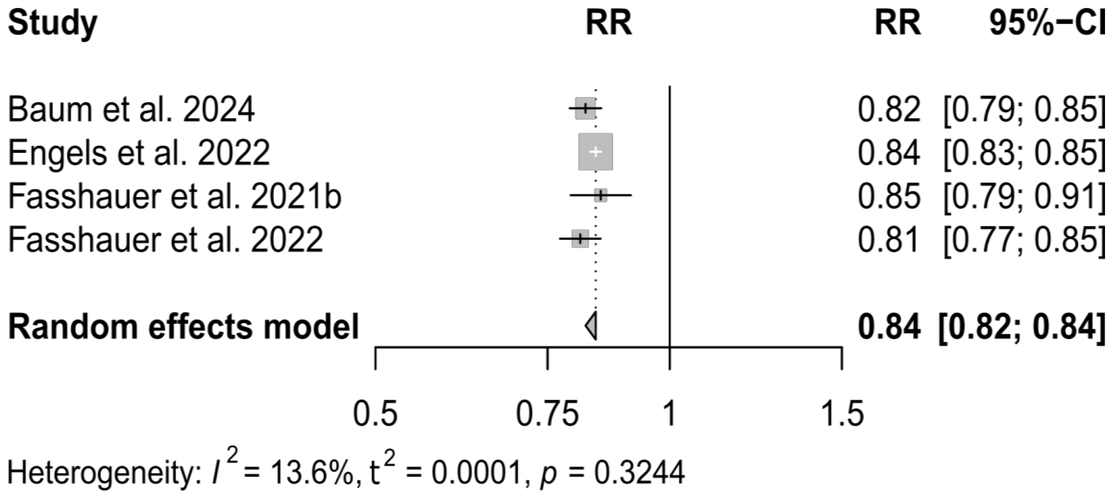

Supplement D - Notaufnahme

a Notaufnahme - 1. Lockdown-Phase - alle Diagnosen

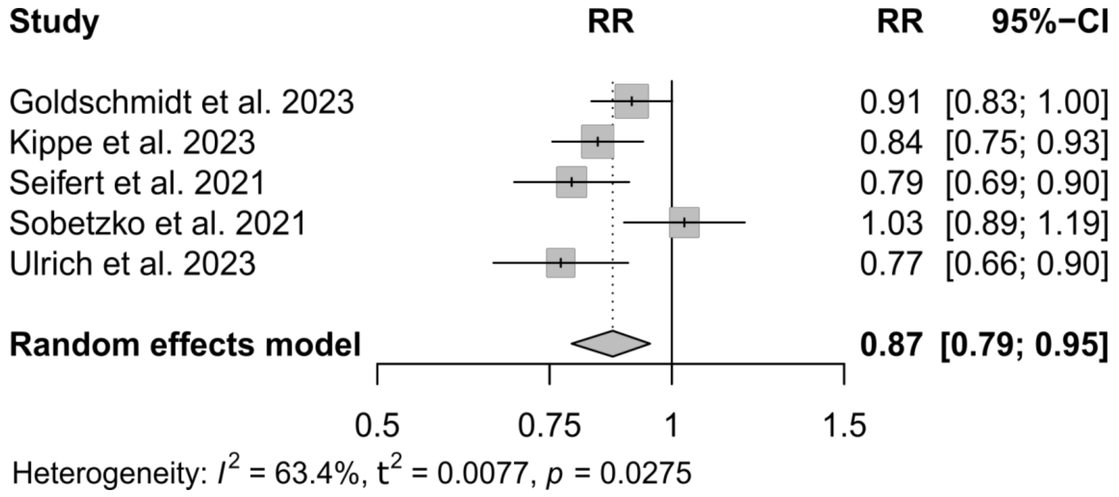

b Notaufnahme - 1. Lockdown-Phase F1

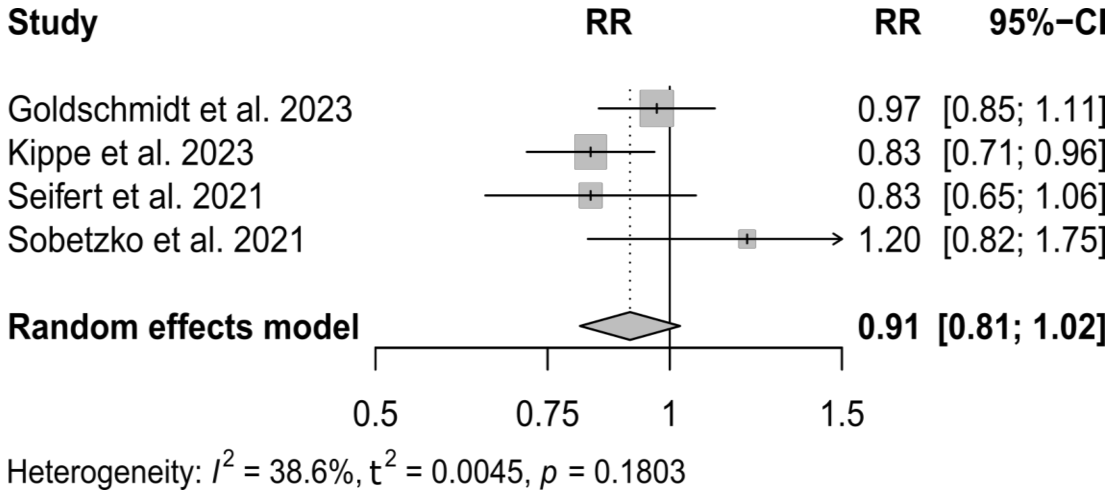

c Notaufnahme –1. Lockdown-Phase F2

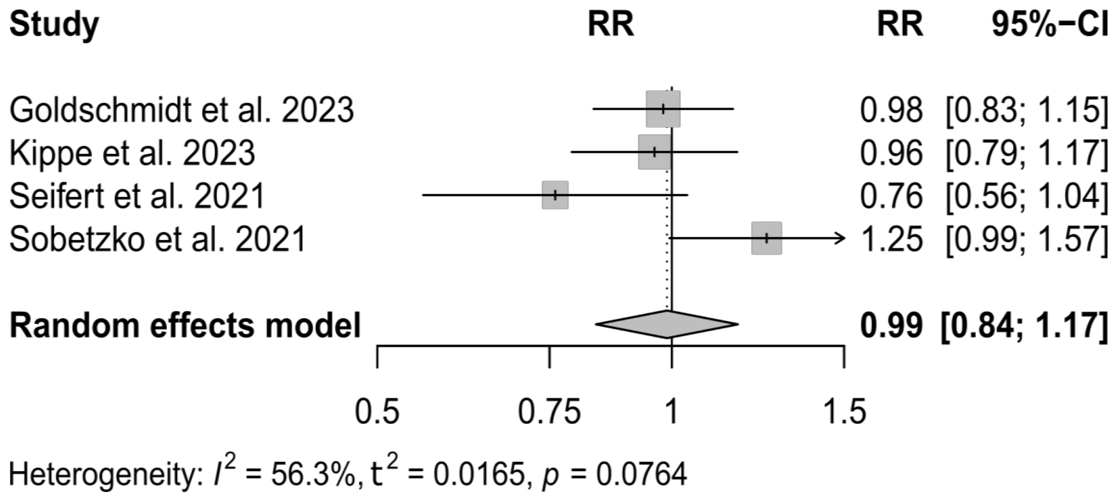

d Notaufnahme –1. Lockdown-Phase F3

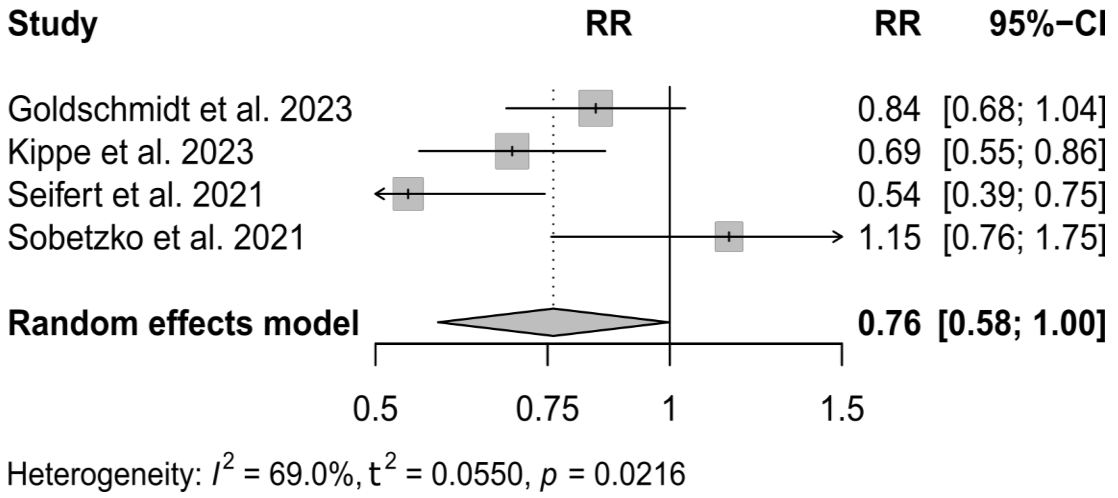

e Notaufnahme 1. Lockdown-Phase F4

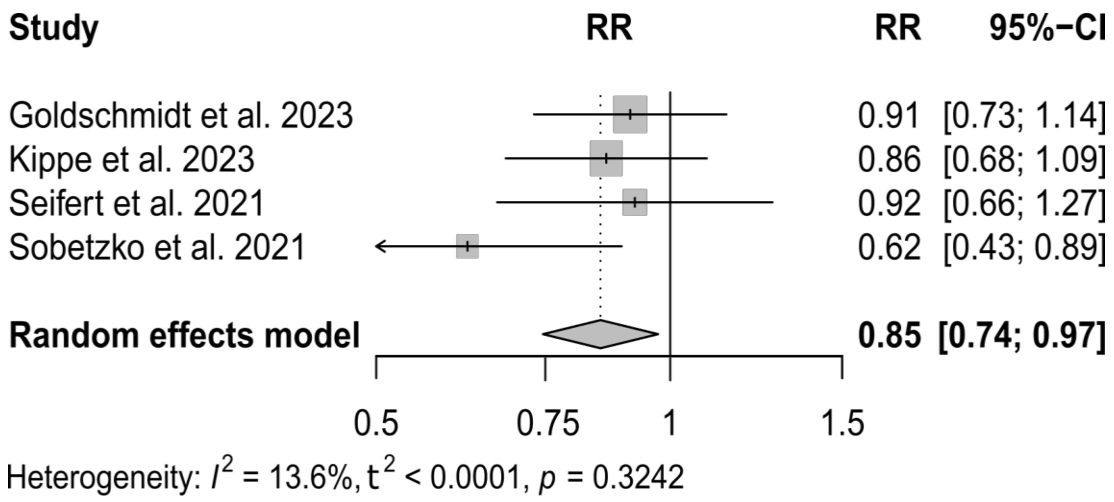

f Notaufnahme - 1. Lockdown-Phase F6

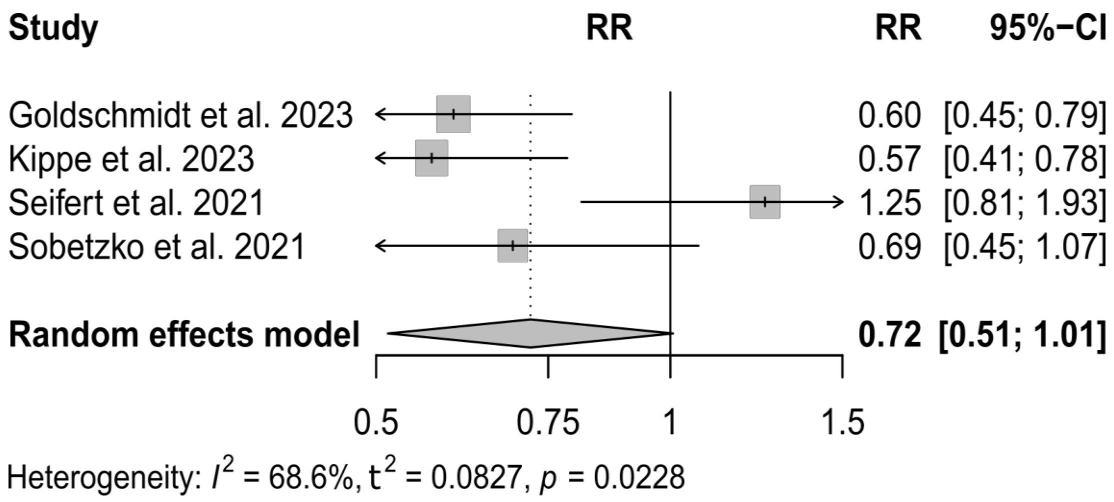

g Notaufnahme 2. Lockdown-Phase alle Diagnosen

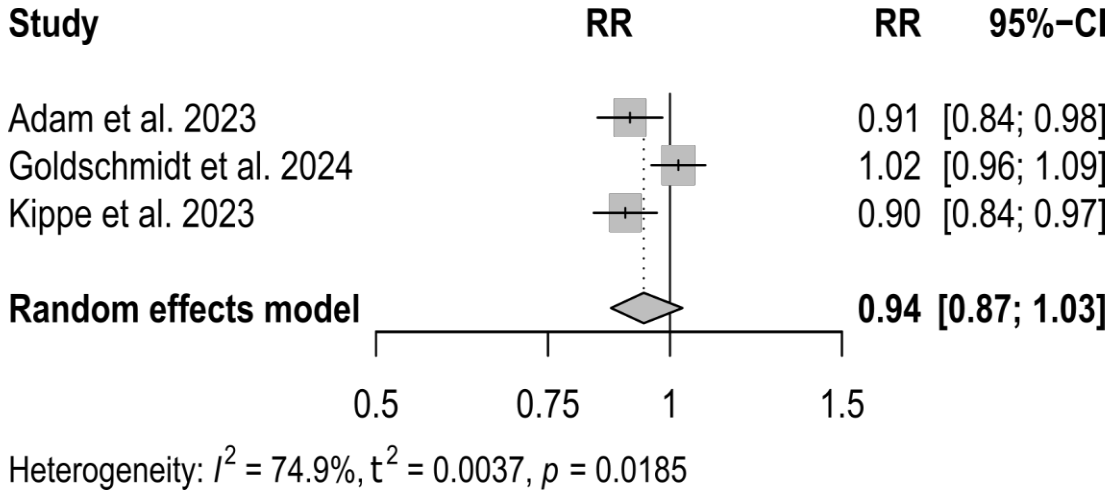

Supplement: Supplementary file 2 — Supplement [file 115_2025_1812_MOESM2_ESM.pdf]
